# Ipriflavone From *Aquilaria malaccensis* Lam. Exosome‐Like Nanoparticles Targets Prolyl Hydroxylase Domain Protein 2 (PHD2) to Enhance Hypoxia‐Inducible Factor‐α (HIF‐α) Hydroxylation Thereby Alleviating Hypoxia‐Induced Gastrointestinal Mucosal Ferroptosis

**DOI:** 10.1002/mco2.70722

**Published:** 2026-04-09

**Authors:** Dezhi Wang, Xingchen Liao, Yilin Wang, Xuexin Wang, Heng Zhang, Jie Zeng, Mingjie Zhang, Xin Wang, Fangli Ren, Yinyin Wang, Meng Li, Wenchen Wang, Qing Lin, Lingyun Gu, Zhijie Chang, Jianqiu Sheng

**Affiliations:** ^1^ Medical School of Chinese PLA Chinese PLA General Hospital Beijing China; ^2^ Department of Gastroenterology The Seventh Medical Center of Chinese PLA General Hospital Beijing China; ^3^ Department of Urology The Second Affiliated Hospital, School of Medicine South China University of Technology Guangzhou China; ^4^ State Key Laboratory of Membrane Biology School of Medicine Institute of Precision Medicine Tsinghua University Beijing China; ^5^ School of Medicine Nankai University Tianjin China; ^6^ Department of Anesthesiology and Critical Care Medicine School of Medicine Johns Hopkins University Baltimore Maryland USA; ^7^ Senior Department of TCM The Sixth Medical Center of Chinese PLA General Hospital Beijing China

**Keywords:** *Aquilaria malaccensis* Lam. exosome‐like nanoparticles (AELNs), ferroptosis, hypoxia, hypoxia‐inducible factor‐α (HIF‐α), ipriflavone, prolyl hydroxylase domain protein 2 (PHD2)

## Abstract

The major challenge in the clinical treatment of gastrointestinal mucosal injury caused by high‐altitude hypoxic environments lies in its unclear underlying mechanisms. In the previous study, we found that hypoxia‐induced gastric and small intestinal damage was mainly attributable to ferroptosis mediated by hypoxia‐inducible factor‐α (HIF‐α; mainly HIF‐1α and HIF‐2α). Both plant exosome‐like nanoparticles and *Aquilaria malaccensis* Lam. have been reported to have antioxidant properties. In the present study, orally delivered *A. malaccensis* Lam. exosome‐like nanoparticles (AELNs) reduced HIF‐1α expression and alleviated gastric and small intestinal mucosal ferroptosis induced by hypoxia. We analyzed the compositions of AELNs and hypothesized that ipriflavone was the effector component, as it showed the highest abundance of metabolites. Subsequent experiments demonstrated that ipriflavone downregulated polyunsaturated fatty acid‐phospholipids, NADPH oxidase 4 (NOX4), and arachidonate 5‐lipoxygenase (ALOX5) by inhibiting HIF‐α, consequently alleviating hypoxia‐induced gastric and small intestinal mucosal ferroptosis. Ipriflavone was found to inhibit HIF‐α by targeting prolyl hydroxylase domain protein 2 (PHD2) to induce it to hydroxylate HIF‐α. This study highlights that ipriflavone, a potent HIF‐α inhibitor, significantly ameliorates the gastric and small intestinal mucosal damage caused by hypoxia and has promise in clinical applications for treating disorders characterized by high levels of HIF‐α.

## Graphical abstract

Ipriflavone (Ipr), an active component of *Aquilaria malaccensis* Lam. exosome‐like nanoparticles (AELNs), significantly reduces hypoxia‐inducible factor‐α (HIF‐α) expression by enhancing the prolyl hydroxylase domain protein 2 (PHD2)‐mediated hydroxylation of HIF‐α, thereby downregulating HIF‐α‐induced polyunsaturated fatty acid‐phospholipids (PUFA‐PLs), NADPH oxidase 4 (NOX4), and arachidonate 5‐lipoxygenase (ALOX5) to ameliorate the gastric and small intestinal mucosal cellular ferroptosis caused by hypoxia.

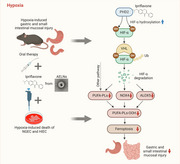

## 1 Introduction

Tens of millions of people worldwide travel annually to high‐altitude regions (> 2500 m) for short‐term work, tourism, and pilgrimage. Exposure to hypoxic environments of high‐altitude plateaus (> 2500 m) often triggers gastrointestinal distress, manifesting as abdominal pain, indigestion, nausea, diarrhea, peptic ulcers, and gastrointestinal bleeding. These disorders are mechanistically linked to hypoxia‐induced oxidative stress, in which the gastric and intestinal barrier integrity is compromised [1, 2]. Current therapeutic approaches remain limited to symptom management, as effective preventive and therapeutic strategies have not yet been developed. Consequently, the development of mechanism‐based therapeutics for high‐altitude hypoxic gastrointestinal disorders constitutes a critical research imperative.

The ability of cells to sense and adapt to hypoxic environments is principally governed by hypoxia‐inducible factor (HIF). HIF represents a family of heterodimeric transcription factors with an O_2_‐regulated HIF‐α subunit (mainly HIF‐1α and HIF‐2α) and a structurally expressed HIF‐1β subunit [3]. During acute hypoxia, stabilized HIF‐α activates transcription of adaptive response genes [4]. In contrast, chronic hypoxic exposure promotes HIF‐α‐mediated cell death [5]. In addition to HIF‐α, the prolyl hydroxylase domain protein family (PHD1–3) is a critical oxygen‐sensing system that regulates HIF‐α stability [6]. HIF‐α undergoes hydroxylation by PHDs, followed by rapid ubiquitination mediated by Von Hippel–Lindau (VHL) that ultimately leads to proteasomal degradation. PHD2 is responsible for most of the hydroxylation activity related to HIF‐α and is considered the principal HIF‐α‐related PHD [7, 8].

Ferroptosis is an iron‐dependent type of programmed cell death characterized by peroxidation of polyunsaturated fatty acid‐containing phospholipids (PUFA‐PLs) and subsequent plasma membrane rupture when the cellular antioxidant system can no longer compensate [9, 10]. The influence of hypoxia and HIF signaling on ferroptosis varies considerably across different cell types: hypoxia often inhibits tumor cell ferroptosis while promoting normal cell ferroptosis [11]. By downregulating HIF‐1α and HIF‐2α, our prior study showed that hypoxia‐triggered ferroptosis in gastric and small intestinal mucosal cells was alleviated through the inhibition of two key effector enzymes: NADPH oxidase 4 (NOX4), a member of the NOX enzyme family, that is, primarily responsible for producing reactive oxygen species (ROS), and arachidonate 5‐lipoxygenase (ALOX5), an enzyme in the lipoxygenase family that generates lipid peroxides [12, 13, 14]. It should be noted that the gastrointestinal mucosal damage induced by hypoxia does not include the esophagus and large intestine, which may be attributable to their relatively greater tolerance to hypoxic conditions [14, 15].

*Aquilaria malaccensis* Lam., an Indo‐Malaysian species of the *Aquilaria* genus, is recognized as a valuable resinous plant due to its antioxidant and anti‐antibacterial properties [16]. Multiple studies have reported that various substances isolated from agarwood also exhibit antioxidant effects [17]. In addition, plant‐derived exosome‐like nanoparticles (PELNs), which contain metabolites, RNAs, and proteins [18], have demonstrated therapeutic potential by modulating intestinal microbiota and repairing mucosal damage [19, 20]. Ipriflavone, an isoflavone with antioxidant and anti‐inflammatory properties [21, 22, 23], is clinically used for the treatment and prevention of osteoporosis in postmenopausal women [24]. It has been reported that ipriflavone functions as an antioxidant in rat models of Alzheimer's disease [23, 25].

In this study, we investigated the effects of *A. malaccensis* Lam. exosome‐like nanoparticles (AELNs) on hypoxia‐induced ferroptosis in gastric and small intestinal mucosa and identified ipriflavone as an active component of AELNs. Mechanistically, we revealed that ipriflavone significantly reduced HIF‐α expression by increasing PHD2‐mediated hydroxylation of HIF‐α, thereby downregulating HIF‐α‐triggered PUFA‐PLs, NOX4, and ALOX5 to alleviate hypoxia‐induced ferroptosis in the gastric and small intestinal mucosa.

## 2 Results

### 2.1 AELNs Relieved Gastric and Small Intestinal Mucosal Injury in Mice Subjected to Hypoxic Conditions

**FIGURE 1:**
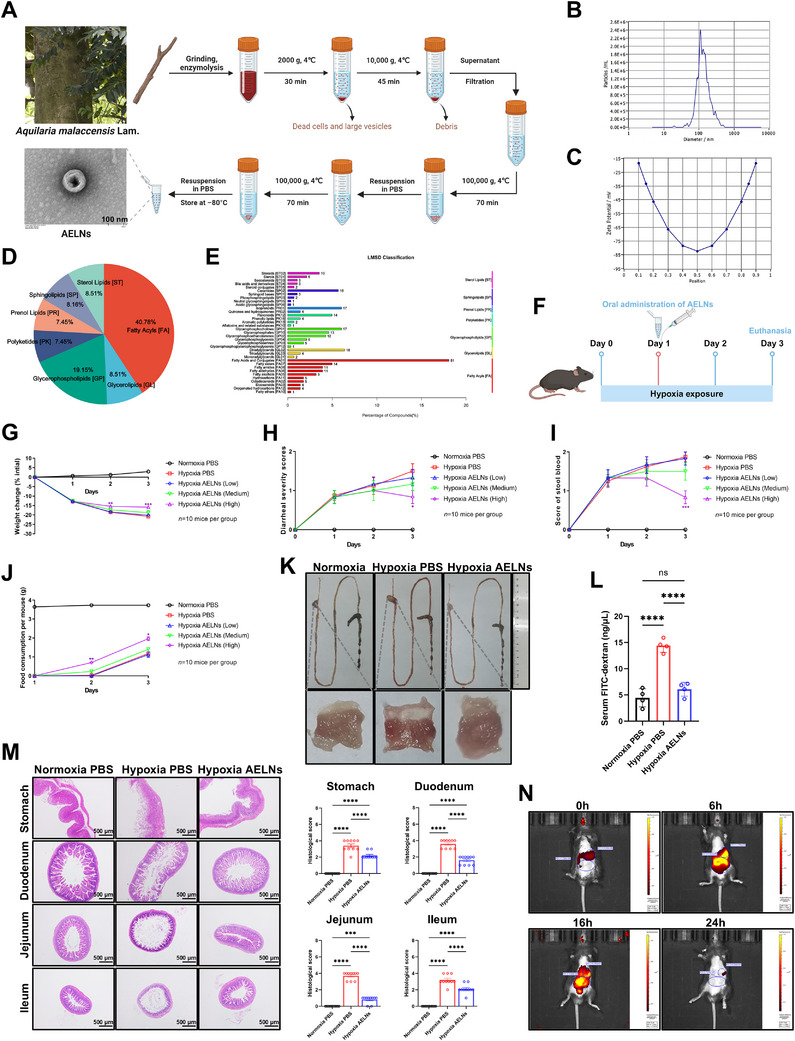
*Aquilaria malaccensis* Lam. exosome‐like nanoparticles (AELNs) relieved gastric and small intestinal mucosal injury in hypoxia‐exposed mice. (A) Flowchart of the AELN isolation process and representative electron micrographs of AELNs. The particle size (B) and surface charge (C) of the AELNs. The lipid composition of AELNs at the class (D) and subclass (E) levels, as determined by liquid chromatography‐mass spectrometry (LC‐MS) with reference to the LIPID MAPS Structure Database (LMSD). Mice were placed in a hypoxic chamber system to simulate an altitude of 5000 m above sea level for 3 days (*n* = 10 mice per group). On Day 1, the mice received an oral gavage of either PBS or AELNs at doses of 1 × 10^4^, 5 × 10^4^, or 1 × 10^5^ particles. All mice were euthanized on Day 3. (F) Schematic overview of the treatments administered to the mice. Body weight (G), diarrheal severity scores (H), fecal occult blood scores (I), and food consumption (J) were recorded daily (*n* = 10). (K) Representative macroscopic photographs of the entire gastrointestinal tract (upper) and gastric mucosa (lower). (L) Serum FITC concentration 4 h after FITC‐dextran gavage to assess gastrointestinal permeability (*n* = 4). (M) Representative hematoxylin and eosin (H&E) staining of the stomach, duodenum, jejunum, and ileum (40× magnification, scale bar: 500 µm) with matching histopathologic scores (*n* = 10). (N) Whole‐body fluorescence imaging of mice 0, 6, 16, and 24 h after oral treatment with DiR‐labeled AELNs. The mice received 1 × 10^5^ particles of AELNs (high‐dose AELNs) (K–N). The purple asterisk indicates a significant difference between the AELN‐treated group (1 × 10^5^ particles) and the PBS control group exposed to hypoxia (G–J). Data are presented as mean ± standard error of the mean (SEM). One‐way ANOVA (L, M) and two‐way ANOVA (G–J): ^*^ *p* < 0.05, ^**^ *p* < 0.01, ^***^ *p* < 0.001, and ^****^ *p* < 0.0001; ns, not significant.

AELNs were extracted via ultracentrifugation, and their morphology was observed by transmission electron microscopy (TEM) (Figure 1A). The particle size and surface charge of the AELNs were 145.8 nm and −83 mV, respectively, as determined by nanoparticle tracking analysis (Figure 1B,C). The lipid composition of the AELNs at the class level, identified via liquid chromatography‐mass spectrometry (LC‐MS) coupled with the LIPID MAPS Structure Database (LMSD), was 40.78% fatty acyls, 19.15% glycerophospholipids, 8.51% glycerolipids, 8.51% sterol lipids, 8.16% sphingolipids, 7.45% prenol lipids, and 7.45% polyketides (Figure 1D). Among the fatty acyls, fatty acids and their conjugates had the highest content, at 51% (Figure 1E). To explore the therapeutic effect of AELNs on hypoxia‐induced gastrointestinal injury, mice were gavaged with phosphate buffered saline (PBS) or various concentrations of AELNs under hypoxic conditions (Figure 1F). The results indicated that high‐dose AELNs (1 × 10^5^ particles) significantly alleviated hypoxia‐induced weight loss, loose stools, fecal occult blood, and low food consumption (Figure 1G–J). Under normoxic conditions, the same high dose of AELNs did not exert any detectable effect on gastrointestinal parameters (Figure S1). Upon macroscopic examination, redness was observed along the entire digestive tract and in the gastric mucosa following hypoxic exposure (Figure 1K). Permeability assay showed that AELN treatment partially reversed the increased gastrointestinal permeability induced by hypoxia (Figure 1L). Furthermore, histological analysis indicated that hypoxia caused the loss and shedding of mucosal cells. This was accompanied by villus structure disorganization, shallow crypts in the small intestinal mucosa, and erosion/thinning of both gastric and intestinal linings, which were clearly evident. Administration of AELNs significantly attenuated this hypoxia‐induced mucosal barrier disruption (Figure 1M). The biodistribution of DiR‐labeled AELNs was assessed using a whole‐body fluorescence imaging system. The distribution of DiR‐labeled AELNs shifted from predominant gastrointestinal accumulation at 6 h to near‐complete elimination by 24 h (Figure 1N).

### 2.2 Orally Administered AELNs Ameliorated Gastric and Small Intestinal Mucosal Ferroptosis Caused by Hypoxia in Mice

**FIGURE 2:**
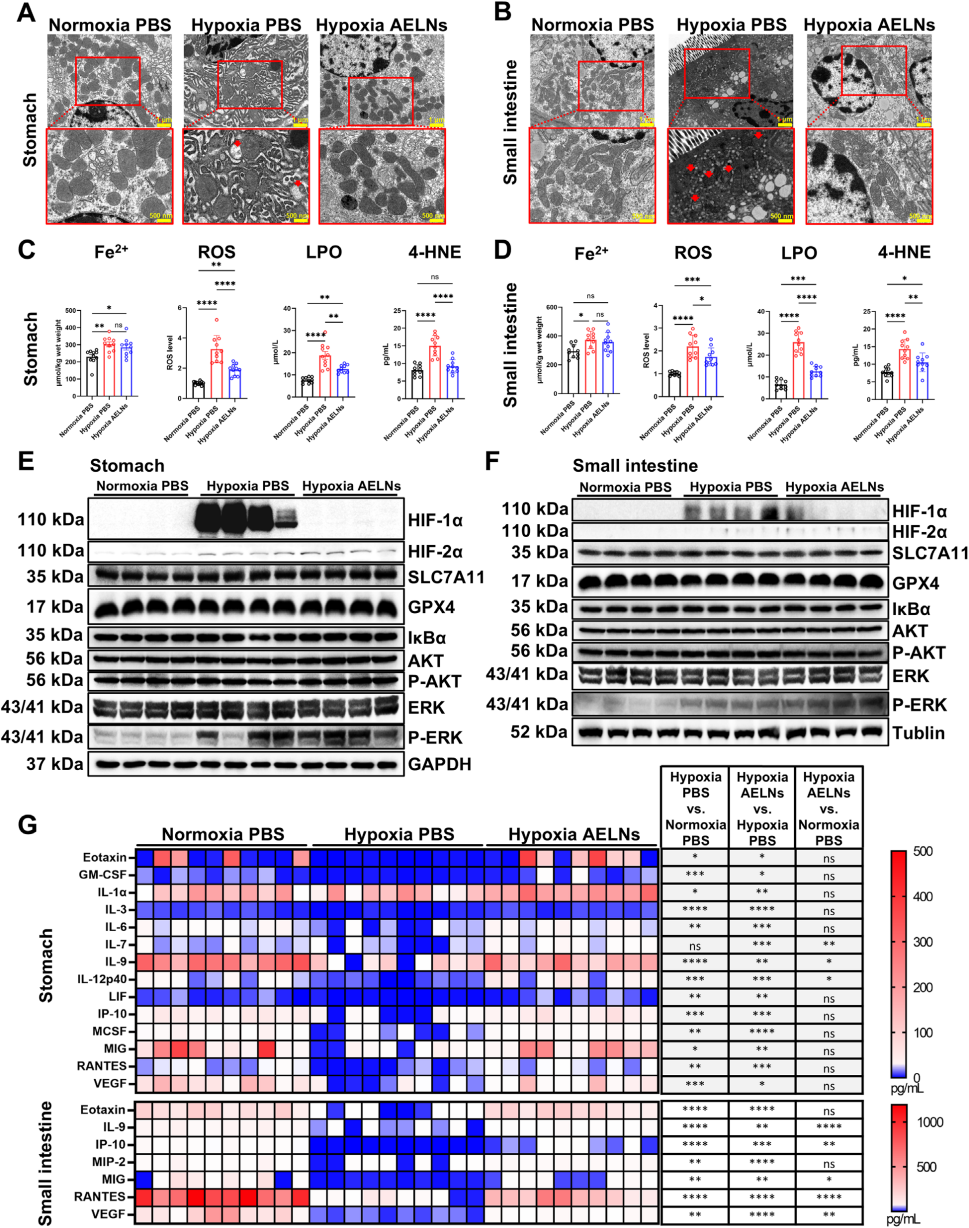
Oral administration of AELNs ameliorated gastric and small intestinal mucosal ferroptosis caused by hypoxia in mice. The mice were housed in a vented hypoxic chamber for 3 days (*n* = 10 mice in each group). They received PBS or AELNs (1 × 10^5^ particles) via oral gavage on Day 1 and were euthanized on Day 3. Representative photographs showing the microstructures of the murine gastric (A) and small intestinal mucosal cells (B) under transmission electron microscopy (TEM). The red arrows indicate shrunken mitochondria (scale bar: overview, 1 µm; inset, 500 nm). Quantification of Fe^2+^, reactive oxygen species (ROS), lipid peroxidation (LPO), and 4‐hydroxynonenal (4‐HNE) of the gastric (C) and small intestinal (D) mucosa (*n* = 10). Western blot analysis of the indicated protein levels in the gastric (E) and small intestinal (F) mucosa assessed by Western blot. (G) Cytokines in the gastric (upper) and small intestinal (lower) mucosa (*n* = 10). Error bars indicate mean ± SEM. One‐way ANOVA: ^*^ *p* < 0.05, ^**^ *p* < 0.01, ^***^ *p* < 0.001, and ^****^ *p* < 0.0001; ns, not significant.

Our previous study showed that ferroptosis contributed to hypoxia‐induced gastric and small intestinal mucosal injury, with TEM revealing ferroptotic mitochondrial changes in PBS‐treated hypoxic mice, including deformed mitochondria, cristae destruction, and an increased membrane density, in both the gastric and small intestinal mucosa [14]. The TEM results in this study also indicated that hypoxic mice treated with PBS exhibited the same ferroptotic mitochondrial changes. However, AELN administration effectively restored the mitochondrial morphology (Figure 2A,B). Consistent with our TEM findings, we found that AELN administration significantly reduced the increased levels of ROS, lipid peroxidation (LPO), and 4‐HNE induced by hypoxia, but it had little effect on Fe^2+^ levels (Figure 2C,D). This indicated that the therapeutic effect of AELNs on ferroptosis did not involve iron regulation. Western blot analysis showed that AELNs significantly reduced the hypoxia‐induced increased level of HIF‐1α but not that of HIF‐2α. Moreover, SLC7A11 and GPX4 levels (the SLC7A11/GPX4 axis is a negative regulator of ferroptosis) did not change among the three groups, indicating that AELNs may have alleviated mucosal ferroptosis by inhibiting HIF‐1α rather than by regulating SLC7A11/GPX4. In our investigation of this hypoxia model, the primary objective was to ascertain the effect of AELNs on HIF‐α. Guided by Kyoto Encyclopedia of Genes and Genomes (KEGG) pathway analysis related to HIF‐α signaling, we examined key proteins from three major upstream pathways to assess whether AELNs influenced HIF‐α through these mechanisms. We found that hypoxia upregulated the ERK pathway without affecting the AKT or NF‐κB pathway. Moreover, AELNs did not affect hypoxia‐induced P‐ERK activation, indicating that AELNs modulated HIF‐α independently of the ERK pathway (Figure 2E,F). Cytokine and chemokine levels were broadly downregulated in the mucosa under hypoxic conditions, as shown by multiplexed enzyme‐linked immunosorbent assay (ELISA). Both of these manifestations were rescued by AELN treatment (Figure 2G). The recovery of cytokine levels by AELNs may have resulted from the inhibition of hypoxia‐induced ferroptosis of immune cells.

### 2.3 AELNs Partially Restored Hypoxia‐Induced Microbial and Metabolic Disorders in the Gastric and Small Intestinal Contents

We also analyzed the effects of AELNs on the microbiota and metabolome in the gastric and small intestinal lumens of the mice exposed to hypoxia. The results revealed that AELNs ameliorated the decline in α‐diversity under hypoxia (Figure S2A,B) and partially restored the microbiota composition, with the most significant recovery in the genera *Ilumatobacter* and *Lachnospiraceae* unclassified in the stomach, and *Kroppenstedtia* and *Enterococcus* in the small intestine (Figure S2C–F). AELN treatment also significantly recovered the hypoxia‐induced decreases in the levels of 3,4‐dihydroxymandelaldehyde and 8‐hydroxyquinoline (stomach) and the levels of 10,11‐dihydroxycarbamazepine, deoxyadenosine, ethyl icosapentate, guanosine, and nervonic acid (small intestine). Moreover, the elevation of palmitoyl‐L‐carnitine (stomach) as well as aspartylglycosamine and calcitriol (small intestine) induced by hypoxia was significantly attenuated by AELN treatment (Figure S3A,B). Correlation analysis between the microbiota and metabolites in the stomach indicated that the microbiota genus *Ilumatobacter* was positively associated with 3,4‐dihydroxymandelaldehyde and 8‐hydroxyquinoline levels but negatively associated with palmitoyl‐L‐carnitine levels. The microbiota genus *Lachnospiraceae* unclassified positively correlated with the levels of both 3,4‐dihydroxymandelaldehyde and N‐formyl‐L‐methionine (Figure S3C). In the small intestine, the microbiota genus *Kroppenstedtia* exhibited a positive correlation with 10,11‐dihydroxycarbamazepine, while the genus *Enterococcus* showed a negative association with ethyl icosapentate (Figure S3D).

### 2.4 Ipriflavone, an Active Component of AELNs, Alleviated Hypoxia‐Induced Ferroptosis in NGEC and HIEC by Inhibiting Both HIF‐1α and HIF‐2α

**FIGURE 3:**
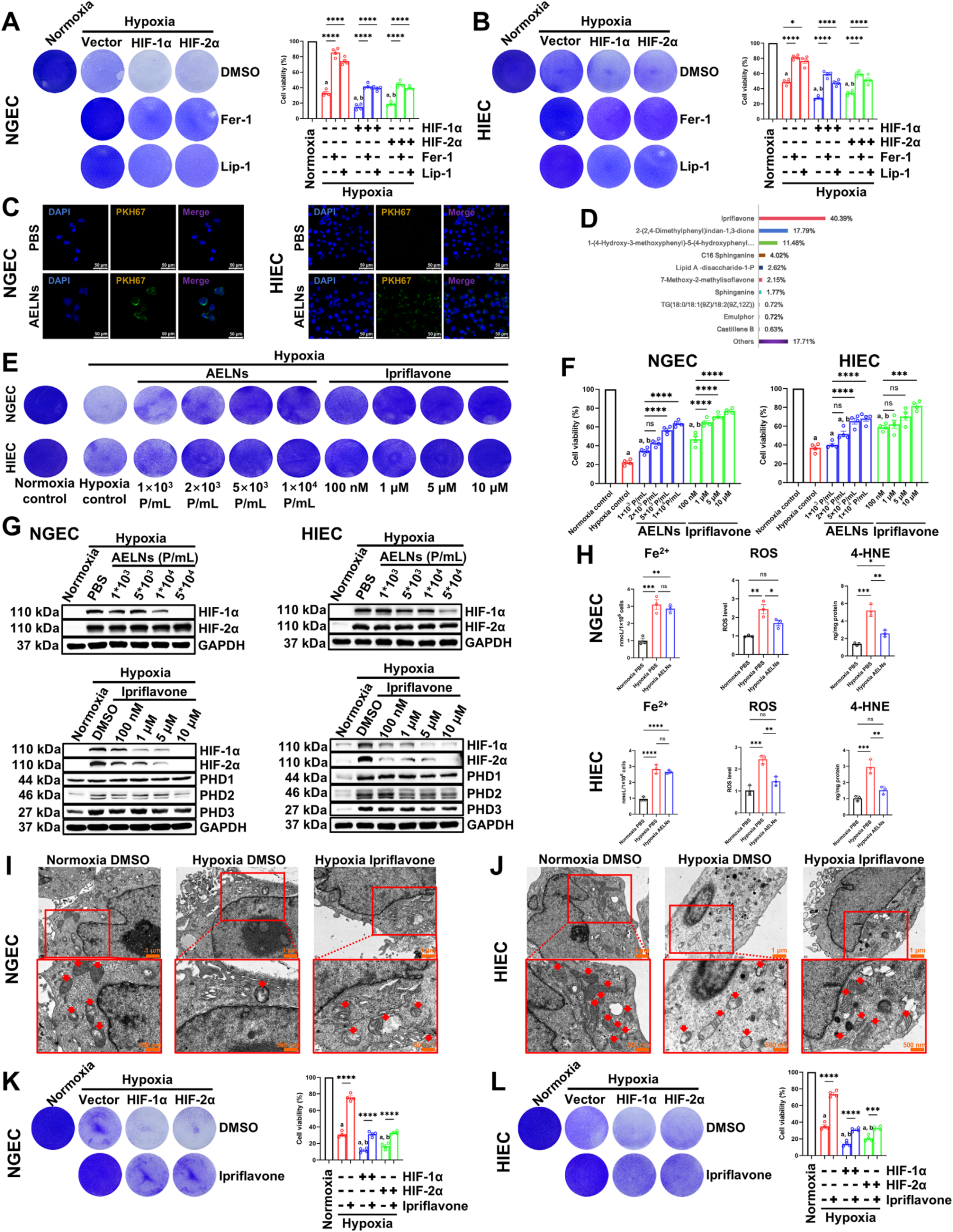
Ipriflavone, an active component of AELNs, alleviated hypoxia‐induced ferroptosis in NGEC and HIEC by inhibiting HIF‐1α and HIF‐2α. NGEC and HIEC were transiently transfected with an empty vector or plasmids containing *HIF‐1α* or *HIF‐2α* for 24 h under normoxic conditions, followed by hypoxia (1% O_2_) for 24 h. The cells were then incubated with dimethyl sulfoxide (DMSO), ferrostatin‐1 (Fer‐1, 5 µM), or liproxstatin‐1 (Lip‐1, 1 µM) for an additional 24 h under hypoxia. Crystal violet staining and corresponding cell viability quantification (*n* = 4) of NGEC (A) and HIEC (B). (C) Uptake of PKH67‐labeled AELNs (1 × 10^4^ p/mL) by NGEC and HIEC after 24 h of incubation, as assessed by confocal microscopy (PKH67, green; DAPI, blue; bar = 50 µm). (D) Metabolomic analysis of the AELN composition. For AELN and ipriflavone treatment, NGEC and HIEC were subjected to hypoxia (1% O_2_) for 24 h, incubated with AELNs (1 × 10^3^, 2 × 10^3^, 5 × 10^3^, or 1 × 10^4^ p/mL), ipriflavone (100, 1, 5, or 10 µM), or a control for another 24 h under hypoxia. (E) Crystal violet staining. (F) Quantification of cell viability (*n* = 4). (G) Western blot analysis of the indicated protein levels. (H) Quantification of Fe^2+^, ROS, and 4‐HNE in NGEC and HIEC (*n* = 3). Representative TEM images of NGEC (I) and HIEC (J). Red arrows indicate mitochondria (scale bar: overview, 1 µm; inset, 500 nm). NGEC and HIEC transfected with an empty vector or plasmids containing *HIF‐1α* or *HIF‐2α* were incubated under normoxic conditions for 24 h, followed by hypoxia (1% O_2_) for 24 h. The cells were then treated with DMSO or ipriflavone and exposed to hypoxia (1% O_2_) for an additional 24 h. Crystal violet staining and corresponding cell viability assessments (*n* = 4) of NGEC (K) and HIEC (L). Experiments were performed in triplicate, with the data showing mean ± SEM. One‐way ANOVA: hypoxia + vector, hypoxia + HIF‐1α, or hypoxia + HIF‐2α versus normoxia control, ^a^ *p* < 0.0001; and hypoxia + HIF‐1α or hypoxia + HIF‐2α versus hypoxia + vector, ^b^ *p* < 0.05 or less, in A, B, K, and L. Hypoxia control, hypoxia + AELNs, or hypoxia + ipriflavone versus normoxia control, ^a^ *p* < 0.0001; and hypoxia + AELNs or hypoxia + ipriflavone versus hypoxia control, ^b^ *p* < 0.05 or less, in F. P/mL indicates particles/mL. The concentration of ipriflavone in H–L was 10 µM. ^*^ *p* < 0.05, ^**^ *p* < 0.01, ^***^ *p* < 0.001, and ^****^ *p* < 0.0001; ns, not significant.

To investigate the contribution of HIF‐α to hypoxia‐induced ferroptosis, *HIF‐1α* or *HIF‐2α* was overexpressed in both cell lines. This overexpression exacerbated hypoxic cell death, as validated by crystal violet staining and CCK‐8 assays. Moreover, the ferroptosis inhibitors ferrostatin‐1 (Fer‐1) and liproxstatin‐1 (Lip‐1) significantly rescued hypoxia‐induced cell death with *HIF‐1α* or *HIF‐2α* overexpression (Figure 3A,B). This confirmed that HIF‐α‐mediated ferroptosis was the dominant form of cell death under hypoxia, consistent with our previous findings [14]. We then conducted further in vitro investigations into the cellular uptake of AELNs, their effects, and their potential active components involved in inhibiting cellular ferroptosis following hypoxia. Confocal microscopy revealed that PKH67‐labeled AELNs with green fluorescence were absorbed by both the normal human gastric epithelial cell line (NGEC) and the normal human small intestinal epithelial cell line (HIEC) (Figure 3C). Untargeted metabolomics was applied to clarify the potential active components in AELNs that alleviated gastric and small intestinal mucosal ferroptosis. Ipriflavone was identified as the major component, accounting for 40.39% of the total metabolites (Figure 3D). Therefore, we hypothesized that ipriflavone was the potential main active component, and we compared the effects of ipriflavone with those of AELNs in hypoxia‐induced ferroptosis. Before further investigation, it was essential to determine whether two other constituents—2‐(2,4‐dimethylphenyl)indan‐1,3‐dione (17.79%) and 1‐(4‐hydroxy‐3‐methoxyphenyl)‐5‐(4‐hydroxyphenyl)‐1,4‐pentadien‐3‐one (11.48%), contributed to the observed activity. Subsequent experiments revealed that under hypoxic conditions, neither of these two compounds exerted significant effects on HIF‐1α, HIF‐2α, or cell viability (Figure S5). Using crystal violet staining and CCK‐8 assays, we observed that both AELNs and ipriflavone enhanced the viability of NGEC and HIEC in a concentration‐dependent manner, with ipriflavone being more effective than AELNs (Figure 3E,F). Western blot analysis further revealed that AELNs attenuated the hypoxia‐induced increase in HIF‐1α levels in a concentration‐dependent manner, while ipriflavone downregulated the hypoxia‐induced increase in the levels of both HIF‐1α and HIF‐2α, indicating a potential mechanism underlying the superior efficacy of ipriflavone compared to AELNs. Neither intervention altered the levels of PHDs (Figure 3G). Thus, high concentrations of ipriflavone (10 µM) were used for our subsequent experiments. Ipriflavone has been reported to function as a non‐steroidal glucocorticoid receptor (GR) antagonist [26], and there may be a mutual regulatory relationship between ipriflavone and GR [27]. Therefore, we examined the effects of ipriflavone on the levels of HIF‐1α and GR in NGEC under both normoxic and hypoxic conditions. Western blot analysis revealed that ipriflavone markedly suppressed HIF‐1α under both normoxic and hypoxic conditions. In contrast, neither hypoxia nor ipriflavone exerted any significant effect on GR expression (Figure S6). Similar to the in vivo AELN treatment results, ipriflavone reduced the hypoxia‐induced elevation of ROS and 4‐HNE (Figure 3H). TEM showed that ipriflavone ameliorated the increased hypoxia‐induced mitochondrial membrane density and cristae damage in both NGEC and HIEC (Figure 3I,J). However, the mechanism through which ipriflavone inhibited HIF‐α to improve cellular ferroptosis was unclear. Rescue experiments further demonstrated that ipriflavone improved cell survival by inhibiting HIF‐α under hypoxic conditions (Figure 3K,L). Based on these results, we concluded that the suppression of hypoxia‐triggered ferroptosis in NGEC and HIEC by ipriflavone was attributable to its inhibition of HIF‐α.

### 2.5 Ipriflavone Ameliorated Hypoxia‐Induced Ferroptosis by Inhibiting HIF‐α‐Mediated Lipid Peroxidation in NGEC and HIEC

**FIGURE 4:**
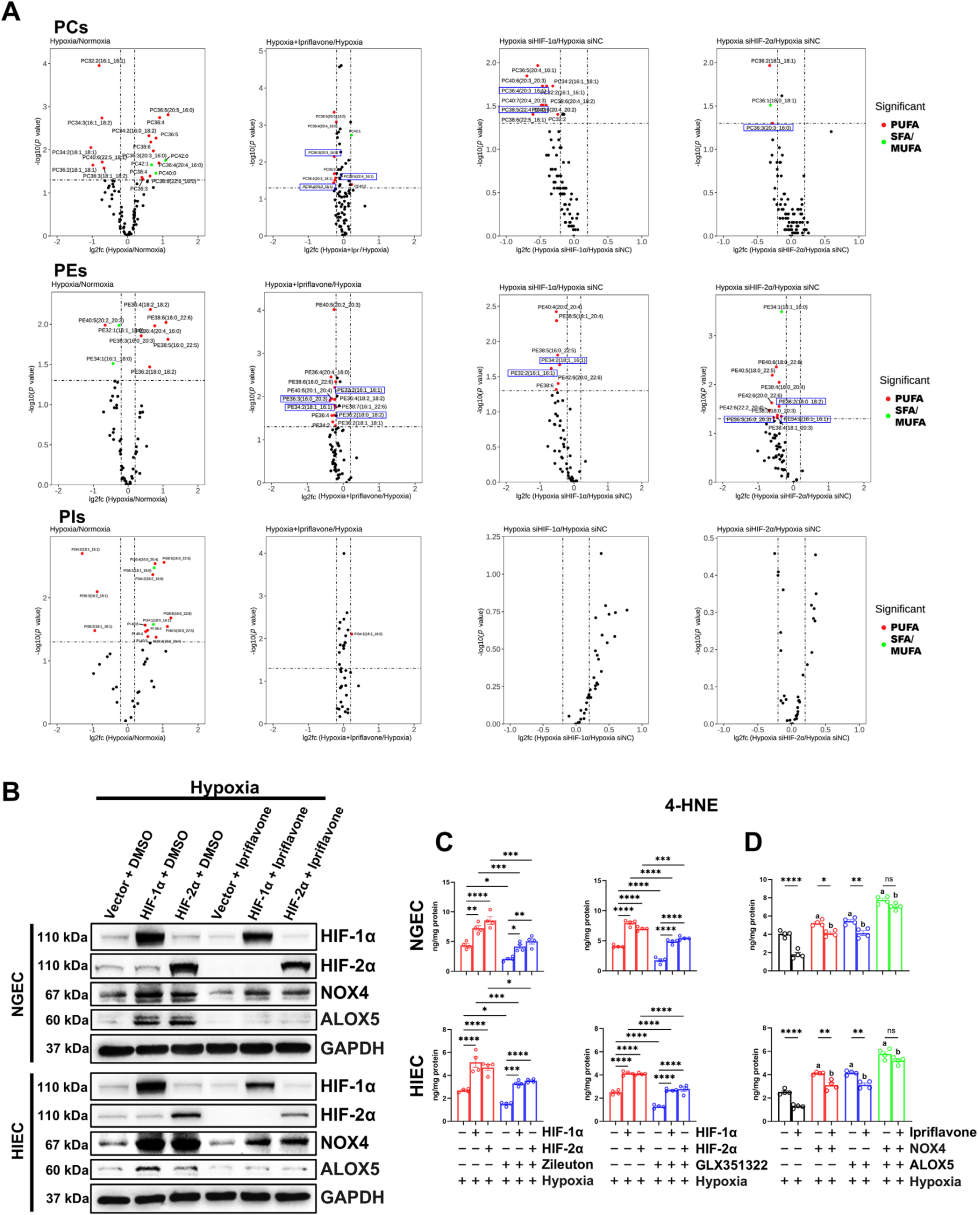
Ipriflavone ameliorated hypoxia‐induced ferroptosis by inhibiting HIF‐α‐triggered PUFA‐PLs, NOX4, and ALOX5 in NGEC and HIEC. Under hypoxia (1% O_2_), HIEC was incubated for 24 h and then treated with ipriflavone (10 µM) for another 24 h. In experiments with siRNA targeting *HIF‐α*, HIEC was transfected with siNC, siHIF‐1α, or siHIF‐2α under normoxia for 24 h and then subjected to hypoxia (1% O_2_) for 48 h. (A) Volcano plots showing the changes in PUFA‐PLs and SFA/MUFA‐PLs, including PC, PE, and PI, between the indicated groups (*n* = 3). The blue boxes indicate the PUFA‐PC and PUFA‐PE with consistent changes after ipriflavone treatment and *HIF‐α* (*HIF‐1α* or *HIF‐2α*) knockdown under hypoxia. NGEC and HIEC were transfected with a control vector or HIF‐1α or HIF‐2α plasmid under normoxia for 24 h, followed by hypoxia (1% O_2_) for 24 h. The cells were then treated with DMSO or ipriflavone (10 µM) under hypoxia for another 24 h. (B) HIF‐1α, HIF‐2α, NOX4, and ALOX5 levels were assessed by Western blot. NGEC and HIEC were transfected with either a blank control vector or plasmids containing *HIF‐1α* or *HIF‐2α* under normoxic conditions for 24 h, followed by exposure to hypoxia (1% O_2_) for 24 h. The cells were then treated with DMSO, zileuton (an ALOX5 inhibitor, 10 µM), or GLX351322 (a NOX4 inhibitor, 10 µM) for an additional 24 h under hypoxic conditions. (C) 4‐HNE levels (*n* = 4) were then assessed using an enzyme‐linked immunosorbent assay (ELISA). NGEC and HIEC were transfected with a blank control vector or plasmids encoding *NOX4* or *ALOX5* for 24 h under normoxia, prior to a 24‐h exposure to hypoxia (1% O_2_). Following this, the cells were incubated with DMSO or ipriflavone (10 µM) for an additional 24 h under hypoxia. (D) 4‐HNE levels (*n* = 4) by ELISA. Representative replicates of three independent experiments are shown in B–D. One‐way ANOVA: hypoxia + NOX4, hypoxia + ALOX5, or hypoxia + NOX4 + ALOX5 versus hypoxia control, ^a^ *p* < 0.05 or less, and hypoxia + NOX4 + ipriflavone, hypoxia + ALOX5 + ipriflavone, or hypoxia + NOX4 + ALOX5 + ipriflavone versus hypoxia + ipriflavone, ^b^ *p* < 0.0001 (in D). ^*^ *p* < 0.05, ^**^ *p* < 0.01, ^***^ *p* < 0.001, and ^****^ *p* < 0.0001; ns, not significant. MUFAs, monounsaturated fatty acids; PCs, phosphatidylcholines; PEs, phosphatidylethanolamines; PIs, phosphatidylinositols; PLs, phospholipids; PUFAs, polyunsaturated fatty acids; SFAs, saturated fatty acids.

We showed that ipriflavone suppressed hypoxia‐induced ferroptosis by inhibiting HIF‐α; however, the specific mechanism behind this remained unclear. Our previous study identified ALOX5 and NOX4 as downstream targets of HIF‐α [14]. Given that (1) high PUFA‐PL levels in cell membranes increase cell susceptibility to ferroptosis while monounsaturated fatty acid (MUFA)‐PLs reduce vulnerability [28], (2) HIF‐α regulates PUFA‐PLs [29], and (3) ALOX5 and NOX4 directly or indirectly oxidize PUFA‐PLs [30], we investigated the mechanism by which ipriflavone alleviated ferroptosis through HIF‐α inhibition by considering the contributions of lipid metabolism, ALOX5, and NOX4. We first quantified key ferroptosis‐associated PLs (phosphatidylethanolamines, PEs; phosphatidylcholines, PCs; phosphatidylinositols, PIs) in each group. Lipidomics analysis revealed PEs and PIs as major contributors of PLs in hypoxia‐induced ferroptosis. Notably, knockdown of *HIF‐1α/HIF‐2α* reduced the levels of PUFA‐PCs and PUFA‐PEs but not those of PUFA‐PIs, indicating that additional regulators beyond HIF‐α may mediate hypoxia‐driven alterations in PUFA‐PLs. Similarly, ipriflavone treatment reduced the levels of PUFA‐PCs and PUFA‐PEs without affecting those of PUFA‐PIs. However, only a limited subset of the PUFA‐PCs and PUFA‐PEs downregulated by siHIF‐1α or siHIF‐2α was also affected by ipriflavone treatment: the PCs PC36:3(20:3_16:0), PC38:5(22:4_16:1), and PC36:4(20:3_16:1) and the PEs PE32:2(16:1_16:1), PE36:3(16:0_20:3), PE34:2(18:1_16:1), and PE36:2(18:0_18:2) (Figure 4A). This suggested that ipriflavone may downregulate PUFA‐PCs and PUFA‐PEs through other regulators of lipid metabolism. We next investigated whether ipriflavone improved ferroptosis by inhibiting the ALOX5‐ and NOX4‐mediated LPO induced by HIF‐α. Under hypoxic conditions, the protein levels of NOX4 and ALOX5, which were mediated by HIF‐1α or HIF‐2α, were reduced following ipriflavone treatment, as demonstrated by Western blot (Figure 4B). Analysis of the LPO biomarker 4‐HNE showed that the additional 4‐HNE triggered by *HIF‐1α*/*HIF‐2α* overexpression was partially reversed by administering zileuton (an ALOX5 inhibitor) or GLX351322 (a NOX4 inhibitor), indicating that HIF‐α inhibited LPO by downregulating ALOX5 and NOX4 levels (Figure 4C). Moreover, restoring NOX4 and ALOX5 levels compromised the ability of ipriflavone to suppress 4‐HNE under hypoxia (Figure 4D). Based on these findings, it was evident that ipriflavone alleviated ferroptosis through HIF‐α inhibition by targeting PUFA‐PLs (PUFA‐PCs and PUFA‐PEs), ALOX5, and NOX4.

### 2.6 Ipriflavone Targeted PHD2 to Enhance HIF‐α Hydroxylation, Thereby Suppressing HIF‐α Expression and Inhibiting HIF‐α‐Mediated Ferroptosis Under Hypoxic Conditions

**FIGURE 5:**
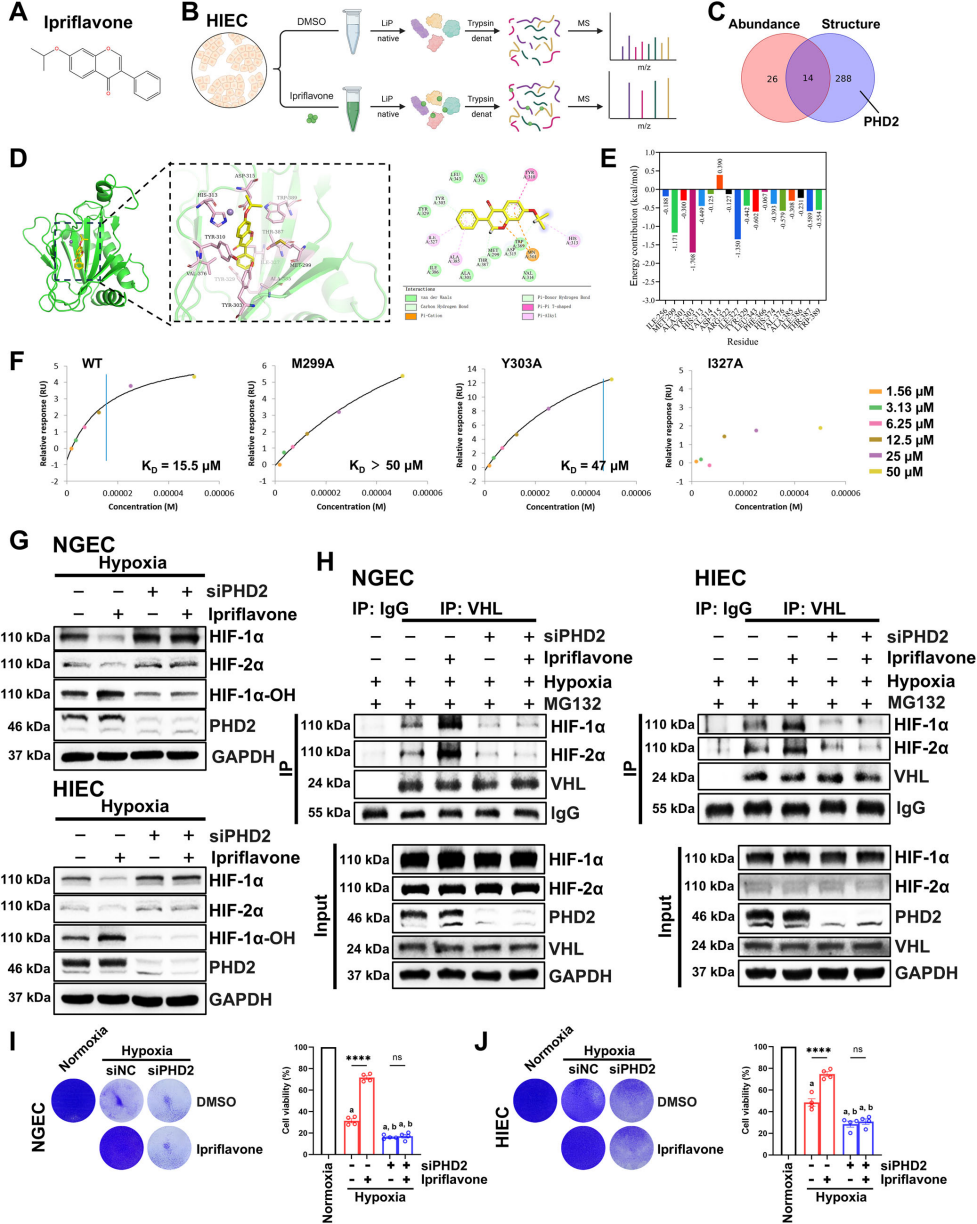
Ipriflavone increased the PHD2‐mediated hydroxylation of HIF‐α to inhibit HIF‐α levels, thereby suppressing the hypoxia‐induced ferroptosis of NGEC and HIEC. (A) Chemical structure of ipriflavone. (B) Schematic illustration of limited proteolysis coupled to mass spectrometry (LiP‐MS). (C) Venn diagram showing the number of proteins with structural and/or abundance‐related changes following ipriflavone treatment (*n* = 3). (D) Predicted binding mode of ipriflavone and PHD2 by AutoDockTools. (E) Per‐residue decomposition of the binding free energy of ipriflavone with PHD2 by molecular dynamics simulation. NGEC and HIEC were transfected with siNC or siPHD2 under normoxia for 24 h and then subjected to hypoxia (1% O_2_) for 24 h. The cells were then incubated with DMSO, ipriflavone, or MG132 for another 24 h under hypoxia. (F) Surface plasmon resonance (SPR) analysis of ipriflavone binding to wild‐type (WT) and various mutant PHD2 proteins. (G) Western blot analysis of the indicated protein levels in NGEC and HIEC. (H) Endogenous VHL immunoprecipitation (IP) was followed by Western blot analysis. Crystal violet staining and corresponding cell viability assessments (*n* = 4) of NGEC (I) and HIEC (J) after ipriflavone administration. The concentration of ipriflavone was 10 µM (in B, C, G–J). Data are representative of three independent experiments (in F–J), and the values represent mean ± SEM. One‐way ANOVA: hypoxia + siNC, hypoxia + siPHD2, or hypoxia + siPHD2 + ipriflavone versus normoxia control, ^a^ *p* < 0.0001, and hypoxia + siPHD2 or hypoxia + siPHD2 + ipriflavone versus hypoxia + siNC, ^b^ *p* < 0.05 or less (in I and J). ^****^ *p* < 0.0001; ns, not significant.

After confirming the mechanism by which ipriflavone inhibited HIF‐α‐mediated ferroptosis, we further investigated the specific mechanism by which HIF‐α was inhibited. The chemical structure of ipriflavone is shown in Figure 5A. The protein targets of ipriflavone were analyzed using limited proteolysis coupled to mass spectrometry (LiP‐MS), which revealed the following potential targets: 302 with structural differences, 40 with abundance differences, and 14 with both structural and abundance differences. PHD2, the primary regulator of HIF‐α, was one of the targets with structural differences (Figure 5B,C).

The binding mode of ipriflavone and PHD2 was predicted using AutoDock Vina. Our results showed that ipriflavone engaged with PHD2 through multiple specific interactions: a pi–donor hydrogen bond with TYR303; carbon–hydrogen bonds with ASP315 and THR387; hydrophobic interactions with ILE327, ALA385, and HIS313; a T‐shaped pi–pi stacking interaction with TYR310; and a pi–cation interaction with an Mn^2+^ ion. In addition, van der Waals forces were observed with other surrounding residues. This multifaceted interaction network enabled ipriflavone to deeply insert into the binding pocket, achieving high‐affinity recognition with a calculated binding free energy of −7.611 kcal/mol (Figure 5D). Molecular docking is typically performed using rigid or semi‐flexible docking, which may not fully account for the conformational flexibility of molecular systems under physiological conditions or their dynamic changes in a solvated environment. To more comprehensively evaluate the binding stability and interaction characteristics of the PHD2–ipriflavone complex, we conducted a 100‐ns all‐atom molecular dynamics simulation based on the docking results (Figure 5E and Figure S7). Binding free energy decomposition identified MET299 (−1.171 kcal/mol), TYR303 (−1.708 kcal/mol), and ILE327 (−1.350 kcal/mol) as primary stabilizers of the PHD2–ipriflavone complex, the significant negative energy contributions of which were essential for overall system stability (Figure 5E). Furthermore, the molecular dynamics simulation results also revealed that the root mean square deviation (RMSD) of the apo form of PHD2 fluctuated between approximately 0.4 and 0.5 nm, with the system reaching equilibrium after about 20 ns. In contrast, the RMSD of the PHD2–ipriflavone complex initially increased rapidly to approximately 0.5–0.65 nm and maintained relatively high fluctuations, indicating that ligand binding induced significant structural rearrangement. The system eventually reached a relatively stable state after 40 ns, suggesting that although ligand binding led to pronounced conformational changes in PHD2, the complex ultimately attained structural equilibrium (Figure S7A). The RMSD of the ligand remained relatively low and stabilized between 0.05 and 0.15 nm, indicating that ipriflavone maintained a stable conformation and binding mode throughout the simulation (Figure S7B). The radius of gyration (Rg) of apo PHD2 was in the range of approximately 1.75–1.82 nm. The Rg of the PHD2–ipriflavone complex was slightly higher, ranging from about 1.78 to 1.85 nm, with a moderate increase observed between 20 and 40 ns. This suggested that ligand binding induced a slight structural expansion in the protein or increased its flexibility, likely resulting from conformational rearrangements in the binding pocket or surrounding regions (Figure S7C). The initial solvent accessible surface area (SASA) was between 120 and 130 nm^2^, which decreased slightly over time and eventually stabilized. The PHD2–ipriflavone complex exhibited slightly higher SASA values, suggesting that ligand binding may have increased the SASA or influenced the conformation of hydrophobic regions. Although minor changes in protein–solvent interaction patterns were observed upon binding, the overall changes were minor (Figure S7D). As shown in Figure S7E, the root mean square fluctuation (RMSF) profiles of the two systems largely overlapped. However, the PHD2–ipriflavone complex showed markedly higher fluctuations in the regions around residues 230–260 and beyond residue 380. These regions, potentially corresponding to flexible loops or regions adjacent to the active site, appeared to exhibit enhanced local flexibility or conformational adaptation upon ligand binding. The Gibbs free energy values (Figure S7F) revealed that the apo form of PHD2 predominantly occupied a single low‐energy well, reflecting limited conformational variability. In contrast, the ipriflavone‐bound system exhibited multiple stable energy basins, characterized by two distinct low‐energy valleys: a compact and an expanded conformation. This indicated that ligand binding altered the energy distribution of the protein and promoted sampling across a broader conformational space, which increased the conformational flexibility and stabilized multiple sub‐states. These findings suggested that ipriflavone may modulate the biological function of PHD2 by regulating its conformational dynamics. The molecular mechanics/Poisson–Boltzmann surface area (MM/PBSA) calculations (Figure S7G) indicated that binding was primarily driven by van der Waals interactions and electrostatic contributions, with additional contributions from non‐polar solvation effects. In contrast, the polar solvation term was unfavorable, indicating a desolvation penalty for polar groups during the binding process. Overall, the binding free energy remained significantly favorable, suggesting that the interaction interface was dominated by hydrophobic and van der Waals interactions, complemented by electrostatic attraction, and that the polar solvation term represented a potential direction for future optimization. Based on the findings from molecular dynamics simulations, three key amino acid residues of PHD2—MET299, TYR303, and ILE327—were individually mutated to alanine. The binding affinity of ipriflavone to wild‐type PHD2 and its mutants was then assessed by surface plasmon resonance (SPR). The SPR results demonstrated that ipriflavone bound to wild‐type PHD2 in a concentration‐dependent manner, with a calculated *K_D_* of 15.5 µM. In contrast, binding with the PHD2 (M299A) mutant was significantly weaker, with *K_D_* > 50 µM. Similarly, the PHD2 (Y303A) mutant also showed a reduced binding affinity, with a *K_D_* of 47 µM (the increase in the relative response may be attributed to conformational changes). For the PHD2 (I327A) mutant, no reliable binding curve could be fitted, indicating a complete loss of detectable binding under the experimental conditions (Figure 5F). Collectively, these data indicated that all three residues, MET299, TYR303, and ILE327, were involved in ipriflavone binding, with ILE327 likely being the most critical residue.

After confirming that ipriflavone targeted PHD2, we further clarified whether ipriflavone inhibited HIF‐α via PHD2. Western blot results showed that, under hypoxic conditions, ipriflavone decreased the levels of HIF‐1α and HIF‐2α and increased the levels of hydroxylated HIF‐1α (no suitable hydroxylated HIF‐2α antibody was available). Furthermore, inhibiting PHD2 increased the levels of HIF‐1α and HIF‐2α and decreased the levels of hydroxylated HIF‐1α. However, once PHD2 was inhibited, ipriflavone failed to regulate HIF‐1α, HIF‐2α, and hydroxylated HIF‐1α, suggesting that ipriflavone inhibited HIF‐α by targeting PHD2 and thus may enhance the PHD2‐mediated hydroxylation of HIF‐α (Figure 5G). Immunoprecipitation (IP) results confirmed that, in the presence of the proteasomal inhibitor MG132 (which ensured the stability of HIF‐α), ipriflavone increased the binding of HIF‐1α and HIF‐2α to VHL, but it failed to exert this effect when PHD2 was inhibited (Figure 5H). Combining the western blot and IP results, we concluded that ipriflavone inhibited HIF‐α levels by increasing PHD2‐mediated hydroxylation. We then inhibited PHD2 and found that ipriflavone was unable to rescue hypoxia‐induced cell death (Figure 5I,J). Combining these results, we determined that ipriflavone promoted HIF‐α degradation by increasing HIF‐α hydroxylation through PHD2, alleviating the ferroptosis driven by HIF‐α in NGEC and HIEC under hypoxic conditions.

### 2.7 Oral Ipriflavone Attenuated Gastric and Small Intestinal Mucosal Damage in Hypoxic Mice

**FIGURE 6:**
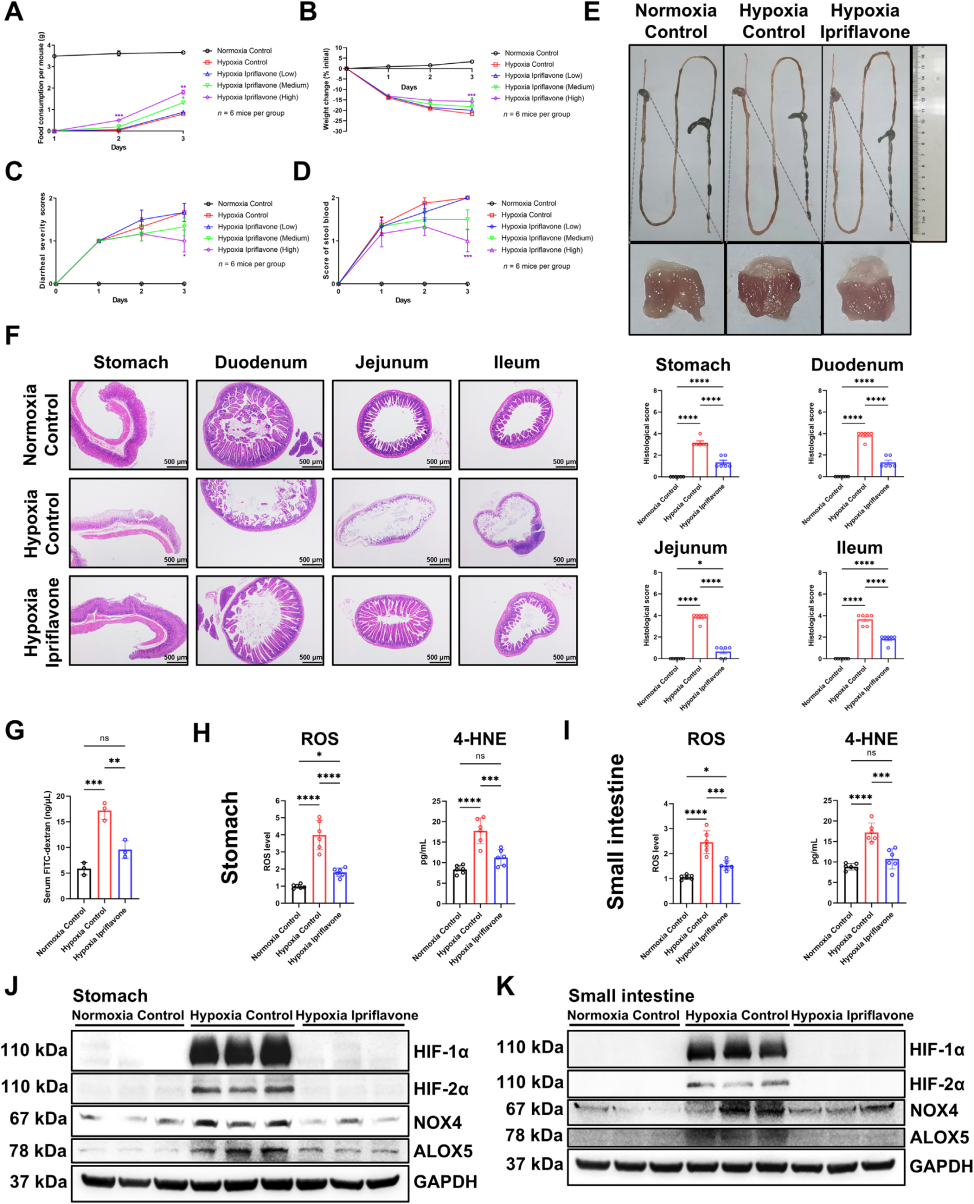
Oral ipriflavone attenuated hypoxia‐induced gastric and small intestinal mucosal ferroptosis in vivo. Mice were placed in a hypoxic chamber for 3 days (*n* = 6 mice per group). The mice were given control solvents or ipriflavone (5, 10, or 15 mg/kg) intragastrically on Day 1 and euthanized on Day 3. Food intake (A), body weight (B), diarrheal severity scores (C), and fecal occult blood test scores (D) of the indicated groups (*n* = 6). (E) Representative images of the gastrointestinal tract and gastric mucosa. (F) Representative H&E‐stained images of murine stomachs and small bowels (40×, scale bar: 500 µm) and corresponding histopathological scores (*n* = 6). (G) Serum FITC concentrations of the indicated groups were used to evaluate gastrointestinal permeability (*n* = 3). ROS and 4‐HNE levels in gastric (H) and small intestinal (I) mucosa (*n* = 6). Protein levels of HIF‐1α, HIF‐2α, NOX4, and ALOX5 in the mucosa of the murine stomachs (J) and small bowels (K). Green or purple asterisks denote significant differences between ipriflavone treatment groups (10 or 15 mg/kg) and the control group subjected to hypoxia (A–D). The mice received 15 mg/kg of ipriflavone (E–K). Data are represented as mean ± SEM. One‐way ANOVA (F–I) and two‐way ANOVA (A–D): ^*^ *p* < 0.05, ^**^ *p* < 0.01, ^***^ *p* < 0.001, and ^****^ *p* < 0.0001; ns, not significant.

Finally, to verify the effects of ipriflavone in vivo, mice were gavaged with a control solvent or varying concentrations of ipriflavone under hypoxic conditions. Compared with the control hypoxia group, the mice in the ipriflavone (high) hypoxia group exhibited improvements in food consumption, body weight, stool consistency, and fecal occult blood (Figure 6A–D). Ipriflavone treatment improved the hypoxia‐induced pathological appearance of the gastrointestinal tract and gastric mucosa and ameliorated gastric and small intestinal mucosa damage (Figure 6E,F). Thus, ipriflavone attenuated hypoxia‐induced gastrointestinal permeability (Figure 6G). Mechanistically, ipriflavone reduced the excessively high production of ROS, 4‐HNE, HIF‐1α, HIF‐2α, NOX4, and ALOX5 induced by hypoxia in the gastric and small intestinal mucosa of the mice (Figure 6H–K), suggesting that ipriflavone improved hypoxia‐induced mucosal ferroptosis by inhibiting HIF‐α, which was consistent with our in vitro results.

## 3 Discussion

Elucidating the pathogenesis of mucosal damage in the stomach and small intestine following hypoxic exposure is a prerequisite for developing effective treatments. Therefore, it is essential to clarify the specific patterns of cell death caused by hypoxia. In this study, we did not observe typical characteristics of apoptosis or necrosis in cells or murine mucosa using TEM. Moreover, the apoptosis inhibitor Z‐VAD‐FMK and the necrosis inhibitor necrostatin‐1 failed to reverse hypoxia‐induced death of NGEC and HIEC in our recent study [31]. In addition, in the present study, hypoxia significantly reduced pro‐inflammatory cytokine levels in gastric and small intestinal mucosa, and our recent findings demonstrated that hypoxia did not alter cleaved caspase‐3 levels in the tissue [31]. Collectively, these responses were not consistent with the characteristics of apoptosis, necrosis, or pyroptosis, with pyroptosis excluded due to its pro‐inflammatory nature. Autophagic activity was observed by TEM in hypoxic cells both in vitro and in vivo. Therefore, the early‐stage autophagy inhibitor 3‐methyladenine (3‐MA) and the late‐stage autophagy inhibitor bafilomycin A1 (BafA1) were administered to determine whether autophagy contributed to the cell death caused by hypoxia. As shown in Figure S4, both 3‐MA and BafA1 exacerbated hypoxia‐induced cell death, suggesting that hypoxia‐induced cell death was not mediated by autophagy. After excluding these cell death modalities, we showed that hypoxia‐induced cell death in gastric and small intestinal mucosa exhibited the defining hallmarks of ferroptosis: elevated Fe^2+^ levels, ROS accumulation, and LPO.

In addition to the AELN concentrations studied here, we previously evaluated three higher doses (1 × 10^6^, 1 × 10^7^, and 1 × 10^8^ particles/mL) that have been commonly used for animal‐derived exosomes in various in vivo models, and we found no therapeutic effects. However, given the physicochemical differences (size and composition) between animal exosomes and PELNs [32], we tested lower concentrations in this study. Remarkably, significant therapeutic benefits were observed at a lower dose (1 × 10^5^ particles/mL).

This study demonstrated that AELNs partially restored hypoxia‐induced microbiota and metabolite disorders. Correlation analysis between the metabolites and microbiota revealed that *Ilumatobacter* was positively associated with 8‐hydroxyquinoline, a metabolite with stronger antioxidant activity than quercetin [33], and negatively associated with palmitoyl‐L‐carnitine, the elevated levels of which impair mitochondrial function and increase ROS production [34]. In addition, AELNs reduced the hypoxia‐induced increased abundance of *Enterococcus*. Mice with necrotizing enterocolitis exhibited higher levels of *Enterococcus* and HIF‐1α compared to controls [35]; however, the relationship between *Enterococcus* and HIF‐1α levels requires further investigation. *Enterococcus faecalis* generates substantial ROS while simultaneously counteracting oxidative stress by inducing antioxidative enzymes that ensure survival under oxidative duress [36]. We observed that the relative abundance of *Enterococcus* exceeded 20% under hypoxia, suggesting an adaptive tolerance to hypoxia‐derived oxidative stress. This enterococcal proliferation may potentiate hypoxia‐induced damage in the gastric and small intestinal mucosa through ROS overproduction. However, the effects of *Enterococcus* were not experimentally validated in this study. Future investigations will examine the role of *Enterococcus* in this pathological context, as well as the specific effects of ipriflavone on microbiota and metabolite profiles.

AELNs downregulated HIF‐1α levels and alleviated hypoxia‐triggered mucosal ferroptosis both in vitro and in vivo. PELNs contain various biomolecular components, including microRNAs, proteins, and metabolites [37, 38]. However, the scarcity of literature on the effects of plant proteins on human cells and physiological systems has hindered the development of a theoretical foundation for further investigations. Therefore, we focused on analyzing microRNAs and metabolites within AELNs to identify the specific bioactive components responsible for decreasing HIF‐1α levels. Our analysis of microRNA‐seq data combined with the RNAhybrid and TargetScan databases found a lack of microRNAs targeting *ALOX5* and *NOX*4. However, we identified specific AELN microRNAs that were predicted to target PHDs, HIF‐1α, and HIF‐2α (BioProject CRA029406). Because of this complex regulatory profile, accurately assessing the net impact of AELN microRNAs on HIF‐1α was challenging. We next aimed to find the potential effector components of AELNs through metabolomic analysis. Ipriflavone was identified as the predominant metabolite in AELNs by metabolomic analysis. Hence, we prioritized ipriflavone as a potential active component in our subsequent experiments. The results demonstrated that ipriflavone ameliorated hypoxia‐induced mucosal ferroptosis by inhibiting HIF‐α. However, AELNs only inhibited HIF‐1α, whereas ipriflavone effectively suppressed both HIF‐1α and HIF‐2α. This indicated that there were additional components in AELNs that regulated HIF‐2α. The different effects of AELNs and ipriflavone on HIF‐2α are potentially attributable to two factors: (1) the influence of PHD‐inhibiting microRNAs, and (2) certain plant‐derived proteins and metabolites within AELNs that may upregulate HIF‐2α levels.

PHDs hydroxylate the proline residues (HIF‐1α, Pro402 and Pro564; HIF‐2α, Pro405 and Pro531) in the oxygen‐dependent degradation domain of the HIF‐α subunit. VHL, in the E3 ubiquitin ligase complex, recognizes the hydroxylated HIF‐α subunits, leading to their rapid degradation through the polyubiquitination‐proteasomal pathway [39]. PHD2 is the primary regulator of HIF‐1α stability [7, 8]. Our results demonstrated that ipriflavone increased the hydroxylation of HIF‐α by PHD2 rather than the levels of PHD2 to downregulate HIF‐α. The specific binding mode between ipriflavone and PHD2 and how the structural changes upon binding enhance PHD2‐mediated HIF‐α hydroxylation therefore merit further investigation, particularly regarding the key amino acid residues (ILE327, MET299, and TYR303).

Ipriflavone, a plant isoflavone, is an osteoporosis drug that has high safety and efficacy [24]. Apart from its anti‐osteoporosis effects, it also exhibits antioxidant and anti‐inflammatory activity [21, 23, 25, 40]. It has been reported that gastrointestinal symptoms may occur following ipriflavone administration. Nevertheless, the gastrointestinal side effects caused by ipriflavone and placebos were similar in the majority of reported randomized controlled trials [24, 41], suggesting that the concomitant prescription of calcium may contribute to the gastrointestinal side effects. Thus, combined with the results of this study, ipriflavone represents a promising clinical treatment for hypoxia‐induced mucosal injury. Ipriflavone has been described as a phytoestrogen [42]. However, in postmenopausal women, ipriflavone fails to exhibit estrogenic effects by activating the estrogen receptor [43, 44]. Ipriflavone prevents bone loss through mechanisms that differ from those of estrogens [42]. This suggests that ipriflavone may be used by men. HIF‐α, a master regulator of basic cellular metabolism and biology, is involved in the development of various tumors and inflammation and their hypoxic microenvironments [45, 46]. Therefore, inhibiting HIF‐α may benefit patients who suffer from these conditions by suppressing proliferation, metabolic reprogramming, angiogenesis, invasion, and metastasis of tumors, or by regulating certain types of overactivated immune cells in inflammation. HIF‐α inhibitors may also be incorporated into existing treatment regimens to improve efficacy [47, 48]. Our findings showed that ipriflavone significantly suppressed HIF‐α levels both in vivo and in vitro, providing promising prospects for treating diseases that involve high HIF‐α levels. Prior to clinical translation, several important issues warrant further investigation. First, the therapeutic effects of ipriflavone observed in murine models of hypoxia‐induced gastric and small intestinal mucosal injury need to be validated in larger animal models that more closely recapitulate human physiology. Second, whether ipriflavone exerts any effects on other organs when administered for the treatment of hypoxia‐induced gastric and small intestinal mucosal injury remains to be determined. Third, the long‐term safety of ipriflavone and its potential drug–drug interactions with medications commonly prescribed in high‐altitude populations should be systematically assessed in future preclinical and clinical studies. Following these necessary preclinical investigations, we plan to conduct clinical trials evaluating several candidate therapeutic agents, including ipriflavone, in populations transitioning from lowland to high‐altitude regions. It is important to note that the effects of HIF‐α vary substantially across different cancers and inflammatory conditions, and its effects are also highly cell‐type‐specific. Therefore, ipriflavone as a treatment should be based on a clear understanding of the mechanisms by which HIF‐α affects specific diseases, and the potential influence of ipriflavone on other targets must also be thoroughly considered.

## 4 Conclusions

Our results clarified the therapeutic effects of AELNs on gastric and small intestinal mucosal ferroptosis induced by hypoxia and identified ipriflavone as an active component of AELNs. Furthermore, we found that ipriflavone significantly decreased the levels of HIF‐α by enhancing PHD2‐mediated hydroxylation, thereby reducing HIF‐α‐induced PUFA‐PL, NOX4, and ALOX5 levels to alleviate gastric and small intestinal mucosal ferroptosis induced by hypoxia. Our findings also highlight that ipriflavone shows promise as a potent HIF‐α inhibitor for use in future clinical practice to treat various diseases that involve HIF‐α dysregulation.

## 5 Materials and Methods

### 5.1 Isolation and Detection of AELNs

*A. malaccensis* Lam. specimens (Yunnan Province, China) were fragmented and enzymatically digested in 20 mL of a solution containing 4% (w/v) cellulase, 2% (w/v) pectinase, and 0.6 mol/L mannitol (pH 5.8) at 50°C for 6 h. The digestate was centrifuged at 2000 × g for 30 min at 4°C to remove dead cells and vesicles, and the supernatant was collected. A subsequent centrifugation (10,000 × g, 45 min, 4°C) was performed to remove debris. The remaining supernatant was filtered through a 0.45‐µm membrane and subjected to ultracentrifugation (100,000 × g, 70 min, 4°C). After discarding the supernatant, the pellet was resuspended in 10 mL of chilled PBS and re‐ultracentrifuged (100,000 × g, 70 min, 4°C). The sediments (AELNs) were collected and resuspended in 150 µL of chilled PBS for subsequent experiments.

AELNs were then added to 300 µL of lysis buffer to precipitate the metabolites. The precipitate was ground, sonicated, centrifuged, and filtered, and the supernatant was collected for metabolomic analysis using a Dionex UltiMate 3000 Rapid Separation LC system (Thermo Fisher Scientific, Waltham, MA, USA) and a Q Exactive hybrid quadrupole Orbitrap mass spectrometer (Thermo Fisher Scientific). A Waters ACQUITY UPLC BEH C8 column (1.7 µm × 2.1 mm × 100 mm) was used to separate the metabolites in both ESI+ and ESI− modes. The mobile phase A contained acetonitrile and water (60/40), and the mobile phase B contained isopropanol and acetonitrile (90/10). Elution was performed at 55°C with a flow rate of 300 µL/min using the following gradient: 0 min, 98% B; 1 min, 98% B; 6 min, 5% B; 7 min, 0% B; 14.5 min, 0% B; 14.6 min, 98% B; and 16 min, 98% B. MS analyses were performed on a Q Exactive hybrid quadrupole Orbitrap mass spectrometer (Thermo Fisher Scientific). The MS/MS scan parameters included a resolution of 17,500, an auto gain control target < 1 × 10^5^, a maximum isolation time of 50 ms, and normalized collision energies of 10, 30, and 60 V. Data were obtained and analyzed using Progenesis QI 2.3 (Waters Corporation, Milford, MA, USA) software and the Human Metabolome (http://www.hmdb.ca), METLIN (https://metlin.scripps.edu), ChemSpider (www.chemspider.com), and LMSD (http://www.lipidmaps.org/data/structure).

### 5.2 Animal Experiments

Male and female C57BL/6J mice (6–8 weeks old) were obtained from Charles River Laboratories (Beijing, China) and were subsequently housed at the specific‐pathogen‐free animal facility of Tsinghua University under a 12‐h–12‐h light–dark cycle. All animal procedures followed the guidelines set by the Animal Care and Use Committee of Tsinghua University (protocol #23‐CZJ1; approval date: March 1, 2023). Following 1 week of acclimatization, age‐ and sex‐matched mice were randomized into five experimental groups for AELN treatment interventions (*n* = 10 per group, with four mice in each group designated for assessing gastrointestinal permeability and thus unavailable for the collection of gastric and small intestinal contents). Mice were subjected to their respective conditions for a total of 3 days, with all treatments administered via oral gavage in a 100 µL PBS vehicle on Day 1. The groups were as follows: a normoxia group, housed under normoxic conditions; a hypoxia group, exposed to hypoxic conditions; and three treatment groups under hypoxia receiving low‐ (1 × 10^4^ particles), medium‐ (5 × 10^4^ particles), or high‐dose (1 × 10^5^ particles) AELNs. In a follow‐up experiment to assess the potential gastrointestinal effects of the optimal dose (1 × 10^5^ particles) under physiological conditions, a separate cohort of mice was administered PBS or high‐dose AELNs via oral gavage on Day 1 and euthanized on Day 3 under normoxia (*n* = 6 per group). In parallel, to evaluate ipriflavone, another set of mice was divided into five groups for ipriflavone treatments (*n* = 6 per group): a normoxia group, gavaged with 100 µL of control solvent (10% dimethyl sulfoxide [DMSO], 40% PEG300, 5% Tween‐80, and 45% PBS); a hypoxia group, gavaged with 100 µL of control solvent; and three hypoxia ipriflavone treatment groups gavaged with ipriflavone (5, 10, or 15 mg/kg; S2422; Selleck, Beijing, China) in 100 µL of solvent. The hypoxia experiments were performed in a ventilated LP‐1500 hypoxic chamber (Yuyan Instruments, Shanghai, China) to simulate a high‐altitude environment of 5000 m above sea level (corresponding to ∼85 mmHg pO_2_) and thereby trigger hypoxia‐mediated gastric and small intestinal injury. Body weight, diarrhea, fecal occult blood, and food intake were monitored daily. Gastric and small intestinal tissue was collected after the mice were euthanized on Day 3 for hematoxylin and eosin (H&E) staining and TEM analysis. Diarrheal severity scores and histological indexes were calculated according to established protocols [14, 49, 50]. Gastric and small intestinal mucosal tissue was collected to perform lipid peroxide analysis, Western blot analysis, and multiplexed ELISA. For 16S rRNA sequencing and metabolomic profiling, contents from the stomach and small intestine were extracted.

### 5.3 Cell Culture and Treatments

NGEC and HIEC, both obtained from Otwo Biotech (Shenzhen, Guangdong, China), were cultured in DMEM supplemented with 10% fetal bovine serum and 1% penicillin–streptomycin (Corning, New York City, NY, USA) in a humidified incubator at 37°C under normoxic conditions. For non‐transfection experiments, NGEC and HIEC were incubated in a GC‐C01 hypoxic incubator (NAWORDE, Beijing, China) and exposed to 1% O_2_ and 5% CO_2_ for 24 h. AELNs (1 × 10^3^, 2 × 10^3^, 5 × 10^3^, or 1 × 10^4^ particles/mL); ipriflavone (100 nM, 1, 5, or 10 µM); 3‐MA (2.5, 5, or 10 mM; Selleck); BafA1 (50, 200, or 500 nM; Selleck); 2‐(2,4‐dimethylphenyl)indan‐1,3‐dione (100 nM, 1 µM, or 10 µM); 1‐(4‐hydroxy‐3‐methoxyphenyl)‐5‐(4‐hydroxyphenyl)‐1,4‐pentadien‐3‐one (100 nM, 1 µM, or 10 µM), or control treatments were then administered, and the cells were incubated for another 24 h under hypoxic conditions. Subsequently, the cells were harvested for crystal violet staining, cell counting kit‐8 (CCK‐8) assays, Western blot analysis, lipid peroxide analysis, TEM, lipidomic analysis, and LiP‐MS. For transfection experiments, HIEC was transfected with siRNAs targeting *HIF‐1α* or *HIF‐2α* (with siNC as a control) under normoxic conditions for 24 h, after which the cells were incubated under hypoxic conditions (1% O_2_) for 48 h. The cells were then collected for lipidomic analysis. For the transfection experiments with reagent treatment, NGEC and HIEC were transfected under normoxic conditions for 24 h with siNC, siPHD2, a pCMV vector plasmid, a pCMV‐HIF‐1α plasmid, a pCMV‐HIF‐2α plasmid, a pCMV‐NOX4 plasmid, or a pCMV‐ALOX5 plasmid. The cells were then exposed to hypoxic conditions (1% O_2_) for 24 h and incubated for another 24 h under hypoxic conditions with DMSO, Fer‐1 (5 µM; S7243; Selleck), Lip‐1 (1 µM; S7699; Selleck), ipriflavone (10 µM), GLX351322 (a NOX4 inhibitor; 10 µM; HY‐100111; MedChemExpress, Shanghai, China), zileuton (an ALOX5 inhibitor; 10 µM; HY‐14164; MedChemExpress), or MG132 (10 µM; S2619; Selleck). The cells were then collected for crystal violet staining, CCK‐8 assays, Western blot analysis, 4‐HNE analysis, or IP. The sequences of the siRNAs used are listed in Table S1.

### 5.4 Assessment of Gastrointestinal Permeability In Vivo

Mice were fasted for 4 h and subsequently administered FITC‐dextran (MW 70,000; MedChemExpress) via oral gavage at a dose of 600 mg/kg. Serum FITC‐dextran concentrations were quantified fluorometrically 4 h post‐administration.

### 5.5 16S rRNA Sequencing and Metabolomic Analysis of Murine Gastric and Small Intestinal Contents

Fresh gastric and small intestinal contents from the mice were collected in sterile tubes and snap‐frozen in liquid nitrogen, then stored at −80°C for 16S rRNA sequencing, metabolomic profiling, and combined analyses of microbial and metabolomic data, according to a previously described protocol [14].

### 5.6 Distribution of AELNs In Vivo

Mice received a single dose of 1 × 10^5^ particles of DiR‐labeled AELNs in 100 µL of PBS via oral gavage. The distribution of AELNs was tracked by using an Odyssey CLx imaging system from LI‐COR Biosciences (Lincoln, NE, USA), with serial fluorescence images acquired 0, 6, 16, and 24 h post‐administration.

### 5.7 Cellular Internalization of AELNs In Vitro

After a 24‐h incubation with PKH67‐labeled AELNs (1 × 10^4^ particles/mL), NGEC and HIEC were processed for imaging by fixation with 4% paraformaldehyde and examination under a confocal laser scanning microscope (TCS SP8 CARS; Leica, Frankfurt, Hessian, Germany).

### 5.8 TEM

The detailed procedures for TEM sample preparation, imaging, and analysis were performed as described in our previous study [14].

### 5.9 Western Blot

Equal amounts of protein were separated by SDS‐polyacrylamide gels and transferred onto nitrocellulose membranes. The membranes were incubated overnight at 4°C with the following primary antibodies at a dilution of 1:1000: HIF‐1α (ab179483; Abcam, Waltham, MA, USA), HIF‐2α (PA1‐16510; Invitrogen, Carlsbad, CA, USA), HIF‐1α (hydroxyl P564, ab308637; Abcam), PHD1 (ab113077; Abcam), PHD2 (ab226890; Abcam), PHD3 (ab184714; Abcam), SLC7A11 (ab307601; Abcam), GPX4 (ab125066; Abcam), AKT (ab8805; Abcam), P‐AKT (ab38449; Abcam), ERK (ab184699; Abcam), P‐ERK (ab201015; Abcam), IκBα (ab32518; Abcam), NOX4 (A22149; ABclonal, Wuhan, Hubei, China), ALOX5 (A2877; ABclonal), VHL (A21083; ABclonal), and GR (ab183127; Abcam). GAPDH (ab8245; Abcam) or Tublin (ab6160; Abcam) was used at a dilution of 1:10,000. Horseradish enzyme‐labeled secondary antibodies (1:10,000) were then applied. Protein bands were visualized using a gel imaging system (SinSage Technology Co. Ltd., Beijing, China).

### 5.10 IP

Cell lysates were incubated at 4°C overnight with 2 µg of anti‐VHL antibody (PA5‐27322; Invitrogen) in parallel with normal rabbit immunoglobulin (as a control). Next, 30 µL of Protein G Sepharose 4 Fast Flow (17061802; Cytiva, Uppsala, Sweden) was added to the cell lysate and incubated at 4°C with rotation for 2 h. The pellets were then washed four times and eluted with sample buffer for subsequent immunoblotting.

### 5.11 Multiplexed ELISA

Cytokine and chemokine levels in murine mucosa were quantified using a multiplexed ELISA kit (MCYTMAG‐70K‐PX32; MilliporeSigma, Burlington, MA, USA) according to the manufacturer's protocol.

### 5.12 Lipid Peroxide Analysis

ROS, 4‐HNE, and LPO levels were measured using kits from Elabscience (ROS, E‐BC‐K138‐F; 4‐HNE, E‐EL‐0128c; LPO, E‐BC‐K176‐M; Wuhan, Hubei, China).

### 5.13 Crystal Violet Staining

Cells were cultured in 12‐well plates, fixed with 4% paraformaldehyde for 1 h, and stained with 0.1% crystal violet (C0121; Beyotime Biotechnology, Shanghai, China) for 30 min. The cells were washed three times with PBS and then observed and photographed under a scanner.

### 5.14 CCK‐8 Assay

Cells were seeded into 96‐well plates. After treatment, 10 µL of CCK‐8 solution (Dojindo Laboratories, Kumamoto, Japan) was added to each well. Following a 2‐h incubation, the absorbance at 450 nm was measured.

### 5.15 Lipidomic Analysis

Lipids were extracted from HIEC and the composition analyzed according to a previously described protocol [14].

### 5.16 LiP‐MS

Samples were placed in lysis buffer with a protease inhibitor (10% of the lysate). After centrifuging at 14,100 × g for 20 min, the supernatant was collected. The protein concentration was determined using the Bradford method. Each sample was split into a trypsin‐only and LiP sample. Proteinase K was added to the LiP sample at an enzyme/substrate (E:S) ratio of 1:100 (wt/wt) and incubated for 1 min at 25°C. Proteinase K digestion was terminated by heating the samples to 100°C for 5 min before cooling to room temperature. SDC was added to the samples in both the TrP and LiP groups. To reduce cysteine residues, TCEP was added to the samples followed by IAA, and the samples were incubated at room temperature for 60 min in the dark. Lysis buffer was added (m [sample protein]:m [Lys] = 100:1) and the sample was incubated at 37°C for 4 h. The SDC in each sample was then diluted to 1%, and the pH was adjusted between 7 and 9. Trypsin was added (m [sample protein]:m [trypsin] = 100:1) and the sample underwent enzymatic digestion overnight at 37°C. Following digestion, FA was added to adjust the pH of the system to < 3. The sample was centrifuged at 4°C and 16,000 × g for 10 min, and the supernatant was collected and immediately analyzed via MS.

Nanoflow liquid chromatography‐tandem mass spectrometry (LC‐MS/MS) analysis of the tryptic peptides was conducted on a quadrupole Orbitrap mass spectrometer (Q Exactive HF‐X; Thermo‐Fisher Scientific, Bremen, Germany) coupled to an EASY nLC 1200 ultra‐high pressure system (Thermo‐Fisher Scientific) with a nano‐electrospray ion source. Peptides (500 ng) were loaded onto a 25‐cm column (150 µm inner diameter, packed with ReproSil‐Pur C18‐AQ 1.5‐µm silica beads). The peptides were separated using a gradient increasing from 8% to 12% B for 8 min, then from 12% to 30% B for 55 min, and then to 40% B for 12 min, followed by a 14‐min wash at 95% B at 600 nL/min. Solvent A was 0.1% formic acid in water, and solvent B was 80% ACN and 0.1% formic acid in water. The total duration of the run was 90 min. The column temperature was maintained at 60°C using a custom oven. Briefly, the mass spectrometer was operated in the “top‐40” data‐dependent mode, collecting MS spectra in the Orbitrap mass analyzer (120,000 resolution, 350–1500 *m/z* range) with an automatic gain control (AGC) target of 3E6 and a maximum ion injection time of 80 ms. The most intense ions from the full scan were isolated using an isolation width of 1.6 *m/z*. Following higher energy collisional dissociation with a normalized collision energy of 27, MS/MS spectra were collected in the Orbitrap (15,000 resolution) with an AGC target of 5E4 and a maximum ion injection time of 45 ms. Precursor dynamic exclusion was enabled with a duration of 16 s.

All raw files were analyzed using the Proteome Discoverer suite version 2.4 (Thermo Fisher Scientific). The MS2 spectra were searched against the UniProt Mus musculus proteome database (55,260 target sequences downloaded on 2023‐03‐07). The Sequest HT search engine was used with parameters specified for MS2 spectra collected by the Orbitrap as follows: semi‐trypsin, maximum of two missed cleavages, minimum peptide length of 6, fixed carbamidomethylation of cysteine residues (+57.02146 Da), variable modifications of methionine residue oxidation (+15.99492 Da), precursor mass tolerance of 15 ppm, and a fragment mass tolerance of 0.02 Da. A percolator was used to filter peptide spectral matches and peptides to a false discovery rate (FDR) of < 1%. After spectral assignment, the peptides were assembled into proteins and filtered to a final FDR of 1% based on the combined probabilities of their constituent peptides. By default, the top matching protein or “master protein” is the protein with the largest number of unique peptides and the smallest peptide coverage percentage (i.e., the longest protein). Only unique and razor (i.e., parsimonious) peptides were considered for quantification. Gene Ontology (GO) and InterPro (IPRIFLAVONE) analyses were conducted using the InterProScan 5 program against the non‐redundant protein database, and the Clusters of Orthologous Groups (COG) and KEGG databases were used to analyze the protein families and pathways. The probable interacting partners were predicted using the STRING‐db server (http://string.embl.de) based on related species. The enrichment pipeline was used to perform both GO and KEGG enrichment analyses.

### 5.17 Molecular Docking

AutoDock Vina version 1.1.2 (Center for Computational Structural Biology, La Jolla, CA, USA) was used for molecular docking in this study. The protein structure of PHD2 was obtained from the UniProt database, and the structure of the small molecule ipriflavone was downloaded from the PubChem database for docking. Ipriflavone was designated as the ligand and PHD2 as the receptor. PyMOL version 4.3.0 software (https://pymol.org) was used to separate the original ligand and protein structure and to dehydrate and remove organic matter. AutoDockTools (http://mgltools.scripps.edu/downloads) was used to add hydrogen, check the charge balance, assign the AD4 type, calculate Gasteiger charges, and build the protein structure docking grid box. The chemical composition of ipriflavone was used to determine the root, and the appropriate torsional bond of the ligand was then selected in AutoDockTools. The file format of both the protein structure and ipriflavone was converted from PDB to PDBQT in AutoDockTools for further docking. After docking using Vina, the scores of the docking combinations for PHD2 and ipriflavone were calculated, and Pymol and Discovery Studio were used for three‐dimensional and two‐dimensional force analyses and visualization.

### 5.18 Molecular Dynamics Simulation

Following molecular docking, a 100‐ns molecular dynamics simulation was conducted using GROMACS v2022.03 to assess the conformational dynamics and temporal stability of the PHD2–ipriflavone complex. The specific molecular dynamics simulation parameters were consistent with those reported by Hu et al. [51]. The system was first equilibrated using an NVT ensemble for 100 ps at 310 K, followed by a 100‐ps NPT equilibration at 1 bar, with an integration time step of 2 fs. Subsequently, the RMSD, Rg, RMSF, and SASA of the complex were calculated. The Gibbs free energy was calculated using the gmx sham and xpm2txt.py utilities in GROMACS, based on the stable RMSD and Rg values derived from the trajectory. The binding free energy of the PHD2–ipriflavone complex was calculated based on the MM/PBSA, using a gmx_MMPBSA script (based on MMPBSA.py v16.0) and analyzing the stable trajectory extracted from the final 20 ns of the simulation.

### 5.19 SPR

The wild‐type and mutant forms of a PHD2 protein fragment (spanning residues 181–426; the full‐length protein was not used due to difficulties in recombinant expression [52]) were covalently immobilized on a CM7 sensor chip (Cytiva, Wilmington, DE, USA) through standard amine coupling chemistry. Three mutations were introduced—M299A, Y303A, and I327A—corresponding to methionine‐299 to alanine, tyrosine‐303 to alanine, and isoleucine‐327 to alanine substitutions, respectively. Different concentrations of ipriflavone were injected and passed over the functionalized chip surface. Binding interactions between ipriflavone and the immobilized PHD2 proteins induced changes in the SPR, which were monitored in real time and recorded as response units. Quantitative binding kinetics were acquired using a Biacore 8K+ instrument (Cytiva). The sensorgram data were analyzed using Biacore evaluation software, which applied affinity and kinetic models to fit the data based on multiple concentration curves and calculated the corresponding *K_D_* values.

### 5.20 Statistical Analysis

Statistical comparisons were performed using one‐way or two‐way analysis of variance. Statistical significance was defined as *p* < 0.05.

## Author Contributions

**Dezhi Wang**: conceptualization, data curation, formal analysis, investigation, methodology, project administration, resources, software, supervision, validation, visualization, writing – review and editing. **Xingchen Liao**: data curation, investigation, software, validation, visualization, writing – original draft. **Yilin Wang**: data curation, investigation, software, visualization. **Xuexin Wang**: investigation, software, validation, visualization, writing – original draft. **Heng Zhang**: data curation, investigation, software. **Jie Zeng**: resources, software. **Mingjie Zhang**: resources. **Xin Wang**: resources. **Fangli Ren**: resources. **Yinyin Wang**: resources. **Meng Li**: software. **Wenchen Wang**: resources. **Qing Lin**: methodology. **Lingyun Gu**: investigation. **Zhijie Chang**: resources, supervision, writing – review and editing. **Jianqiu Sheng**: conceptualization, funding acquisition, methodology, project administration, resources, supervision. All authors have read and approved the final manuscript.

## Ethics Statement

All animal procedures followed the guidelines set by the Animal Care and Use Committee of Tsinghua University (protocol #23‐CZJ1; approval date: March 1, 2023).

## Conflicts of Interest

The authors declare no conflicts of interest.

## Supporting information




**Supplementary Table S1**. Sequences used for siRNA mediated the suppression of HIF‐1α, HIF‐2α, or PHD2.
**Supplementary Figure S1. Aquilaria malaccensis Lam. exosome‐like nanoparticle (AELN) administration showed no significant effect on the clinical signs of digestion in mice under normoxic conditions**. Mice received PBS or AELNs (1 × 10^5^ particles) via oral gavage on day 1 and were euthanized on day 3 under normoxic conditions (n = 6 per group). Food intake (A), body weight (B), diarrheal severity scores (C), and fecal occult blood test scores (D) of the indicated groups (n = 6). Two‐way ANOVA: ns, not significant.
**Supplementary Figure S2. AELNs partially restored gut microbiota in gastric and small intestinal contents altered by hypoxia**. C57BL/6 mice were housed in a hypoxic chamber for 3 days (n = 6 mice in each group). AELNs (5 × 10^4^ particles) or PBS was administered on day 1, and the mice were euthanized on day 3. The alpha diversity of microbial communities in gastric (A) and small intestinal (B) contents (n = 6). Composition of microbial phyla and genera in gastric (C) and small intestinal (D) contents (n = 6). The relative abundance of significantly altered microbiota in gastric (E) and small intestinal (F) contents (n = 6). Data are presented as the mean ± standard error of the mean (SEM). One‐way ANOVA: *p < 0.05, **p < 0.01, ***p < 0.001, ****p < 0.0001 as indicated; ns, not significant.
**Supplementary Figure S3. The hypoxia‐induced metabolic dysfunction in the stomach and small intestine were partially ameliorated by AELNs**. The relative abundance of significantly different metabolites in gastric (A) and small intestinal (B) contents (n = 6). Association analysis of gut microbiota at the phylum and genus levels with metabolites by Spearman correlation analysis in gastric (C) and small intestinal (D) contents (n = 6). Data are presented as the mean ± standard error of the mean (SEM). One‐way ANOVA: *p < 0.05, **p < 0.01, ***p < 0.001, ****p < 0.0001 as indicated; ns, not significant.
**Supplementary Figure S4. Autophagy inhibitors exacerbated hypoxia‐induced cell death in NGEC and HIEC**. NGEC and HIEC were first exposed to either normoxia or hypoxia (1% O_2_) for 24 h. Following this, the cells were treated with vehicle control, 3‐methyladenine (3‐MA; 2.5 mM, 5 mM, or 10 mM), or bafilomycin A1 (BafA1; 50 nM, 200 nM, or 500 nM) for a further 24 h under the same respective oxygen conditions. (A, C) Crystal Violet staining of NGEC and HIEC. (B, D) Assessment of cell viability in NGEC and HIEC cell (n = 3). Data are shown as the mean ± standard error of the mean (SEM). One‐way ANOVA: Normoxia + 3‐MA or normoxia + BafA1 group vs. normoxia control, ^a^ *p* < 0.05 at least (in B, D), and hypoxia + 3‐MA or hypoxia + BafA1 group vs. hypoxia control group, ^b^ *p* < 0.05 at least (in B, D).
**Supplementary Figure S5. No significant effect of 2‐(2,4‐Dimethylphenyl)indan‐1,3‐dione or 1‐(4‐Hydroxy‐3‐methoxyphenyl)‐5‐(4‐hydroxyphenyl)‐1,4‐pentadien‐3‐one on HIF‐1α, HIF‐2α, or cell viability was observed under hypoxic conditions**. NGEC was subjected to hypoxic conditions (1% O_2_) for 24 h, followed by a further 24‐hour treatment under hypoxia with either a vehicle control, 2‐(2,4‐Dimethylphenyl)indan‐1,3‐dione (100 nM, 1 µM, or 10 µM), or 1‐(4‐Hydroxy‐3‐methoxyphenyl)‐5‐(4‐hydroxyphenyl)‐1,4‐pentadien‐3‐one (100 nM, 1 µM, or 10 µM). (A) Protein expression levels of HIF‐1α and HIF‐2α by western blot analysis. (B) Cell viability of NGEC under the indicated treatments (n = 3). One‐way ANOVA: Hypoxia control, hypoxia + 2‐(2,4‐Dimethylphenyl)indan‐1,3‐dione, or hypoxia + 1‐(4‐Hydroxy‐3‐methoxyphenyl)‐5‐(4‐hydroxyphenyl)‐1,4‐pentadien‐3‐one vs. normoxia, ^a^ *p* < 0.0001 (in B).
**Supplementary Figure S6. Neither hypoxia nor Ipriflavone had a significant effect on glucocorticoid receptor (GR) expression**. Following a 24‐hour exposure to hypoxia (1% O_2_), NGEC was treated with ipriflavone (10 µM) or DMSO for a further 24 h under the same conditions. Protein levels of HIF‐1α and GR were analyzed by western blot.
**Supplementary Figure S7. Molecular dynamics simulation of the PHD2–ipriflavone complex**. (A) Root mean square deviation (RMSD) of the PHD2 backbone in the apo and ipriflavone‐bound states. (B) RMSD of the ipriflavone ligand. (C) Radius of gyration (Rg) of PHD2 in the apo and ipriflavone‐bound states. (D) Solvent accessible surface area (SASA) of PHD2 in the apo and ipriflavone‐bound states. (E) Root mean square fluctuation (RMSF) of PHD2 residues in the apo and ipriflavone‐bound states. (F) Gibbs free energy landscape of the apo PHD2 and the PHD2–ipriflavone complex. (G) Binding free energy of the PHD2–ipriflavone complex.

## Data Availability

The data that support the findings of this study are available from the corresponding author upon reasonable request. All genomic, metabolomic, and proteomic data have been deposited in the CNCB‐NGDC database with the following accession numbers: AELN microRNA data, CRA029406; AELN non‐targeted lipidomics data, OMIX011605; 16S rRNA sequencing data, CRA029317; metabolome data of the gastric contents, OMIX011587; metabolome data of the small intestinal contents, OMIX011563; and LiP‐MS data, OMIX011646.
