# Supplementary material for: Ipriflavone From Aquilaria malaccensis Lam. Exosome‐Like Nanoparticles Targets Prolyl Hydroxylase Domain Protein 2 (PHD2) to Enhance Hypoxia‐Inducible Factor‐α (HIF‐α) Hydroxylation Thereby Alleviating Hypoxia‐Induced Gastrointestinal Mucosal Ferroptosis
Source: MedComm (2020). 2026 Apr 9;7(4):e70722. doi: 10.1002/mco2.70722 (PMC13066503; doi:10.1002/mco2.70722)
Supplement: Supplementary file 1 — Supplementary Table S1. Sequences used for siRNA mediated the suppression of HIF‐1α, HIF‐2α, or PHD2. Supplementary Figure S1. Aquilaria malaccensis Lam. exosome‐like nanoparticle (AELN) administration showed no significant effect on the clinical signs of digestion in mice under normoxic conditions. Mice received PBS or AELNs (1 × 10^5^ particles) via oral gavage on day 1 and were euthanized on day 3 under normoxic conditions (n = 6 per group). Food intake (A), body weight (B), diarrheal severity scores (C), and fecal occult blood test scores (D) of the indicated groups (n = 6). Two‐way ANOVA: ns, not significant. Supplementary Figure S2. AELNs partially restored gut microbiota in gastric and small intestinal contents altered by hypoxia. C57BL/6 mice were housed in a hypoxic chamber for 3 days (n = 6 mice in each group). AELNs (5 × 10^4^ particles) or PBS was administered on day 1, and the mice were euthanized on day 3. The alpha diversity of microbial communities in gastric (A) and small intestinal (B) contents (n = 6). Composition of microbial phyla and genera in gastric (C) and small intestinal (D) contents (n = 6). The relative abundance of significantly altered microbiota in gastric (E) and small intestinal (F) contents (n = 6). Data are presented as the mean ± standard error of the mean (SEM). One‐way ANOVA: *p < 0.05, **p < 0.01, ***p < 0.001, ****p < 0.0001 as indicated; ns, not significant. Supplementary Figure S3. The hypoxia‐induced metabolic dysfunction in the stomach and small intestine were partially ameliorated by AELNs. The relative abundance of significantly different metabolites in gastric (A) and small intestinal (B) contents (n = 6). Association analysis of gut microbiota at the phylum and genus levels with metabolites by Spearman correlation analysis in gastric (C) and small intestinal (D) contents (n = 6). Data are presented as the mean ± standard error of the mean (SEM). One‐way ANOVA: *p < 0.05, **p < 0.01, ***p < 0.001, ****p < 0.0001 as indicat [file MCO2-7-e70722-s001.docx]

**Supplementary materials**

**Ipriflavone from Aquilaria malaccensis Lam. exosome-like nanoparticles targets prolyl hydroxylase domain protein 2 (PHD2) to enhance hypoxia-inducible factor-α (HIF-α) hydroxylation thereby alleviating hypoxia-induced gastrointestinal mucosal ferroptosis**

Dezhi Wang^1, 2#^*****, Xingchen Liao^1, 2#^, Yilin Wang^1, 2#^, Xuexin Wang^1, 2#^, Heng Zhang^1, 2^, Jie Zeng^4^, Mingjie Zhang^2^, Xin Wang^2^, Fangli Ren^3^, Yinyin Wang^3^, Meng Li^3^, Wenchen Wang^5^, Qing Lin^6^, Lingyun Gu^7^, Zhijie Chang^3^*****, and Jianqiu Sheng^1, 2^*****

^1^Medical School of Chinese PLA, Chinese PLA General Hospital, Beijing 100853, China,

^2^Department of Gastroenterology, The Seventh Medical Center of Chinese PLA General Hospital, Beijing 100700, China,

^3^State Key Laboratory of Membrane Biology, School of Medicine, Institute of Precision Medicine, Tsinghua University, Beijing 100084, China,

^4^Department of Urology, The Second Affiliated Hospital, School of Medicine, South China University of Technology, Guangzhou 510180, China,

^5^School of Medicine, Nankai University, Tianjin 300071, China,

^6^Department of Anesthesiology and Critical Care Medicine, School of Medicine, Johns Hopkins University, Baltimore 21287, United States of America,

^7^Senior Department of TCM, The Sixth Medical Center of Chinese PLA General Hospital, Beijing 100048, China.

^#^Co-first authors, contributed equally to this work.

***Corresponding author:**

**Dezhi Wang, MD, PhD**

Medical School of Chinese PLA, Chinese PLA General Hospital, Road Fuxing No. 28, Haidian District, Beijing 100853, China

E-mail: zilv_wang@126.com

Tel: +86-10-66721299

**Zhijie Chang, PhD**

State Key Laboratory of Membrane Biology, School of Medicine, Institute of Precision Medicine, Tsinghua University, Beijing 100084, China

E-mail: zhijiec@mail.tsinghua.edu.cn

Tel: +86-10-62785076, Fax: +86-10-62773624

**Jianqiu Sheng, MD, PhD**

Medical School of Chinese PLA, Chinese PLA General Hospital, Road Fuxing No. 28, Haidian District, Beijing 100853, China

E-mail: shengjianqiu@301hospital.org

Tel: +86-10-66721299

**Supplementary table S1. Sequences used for siRNA mediated the suppression of HIF-1α, HIF-2α, or PHD2**

**
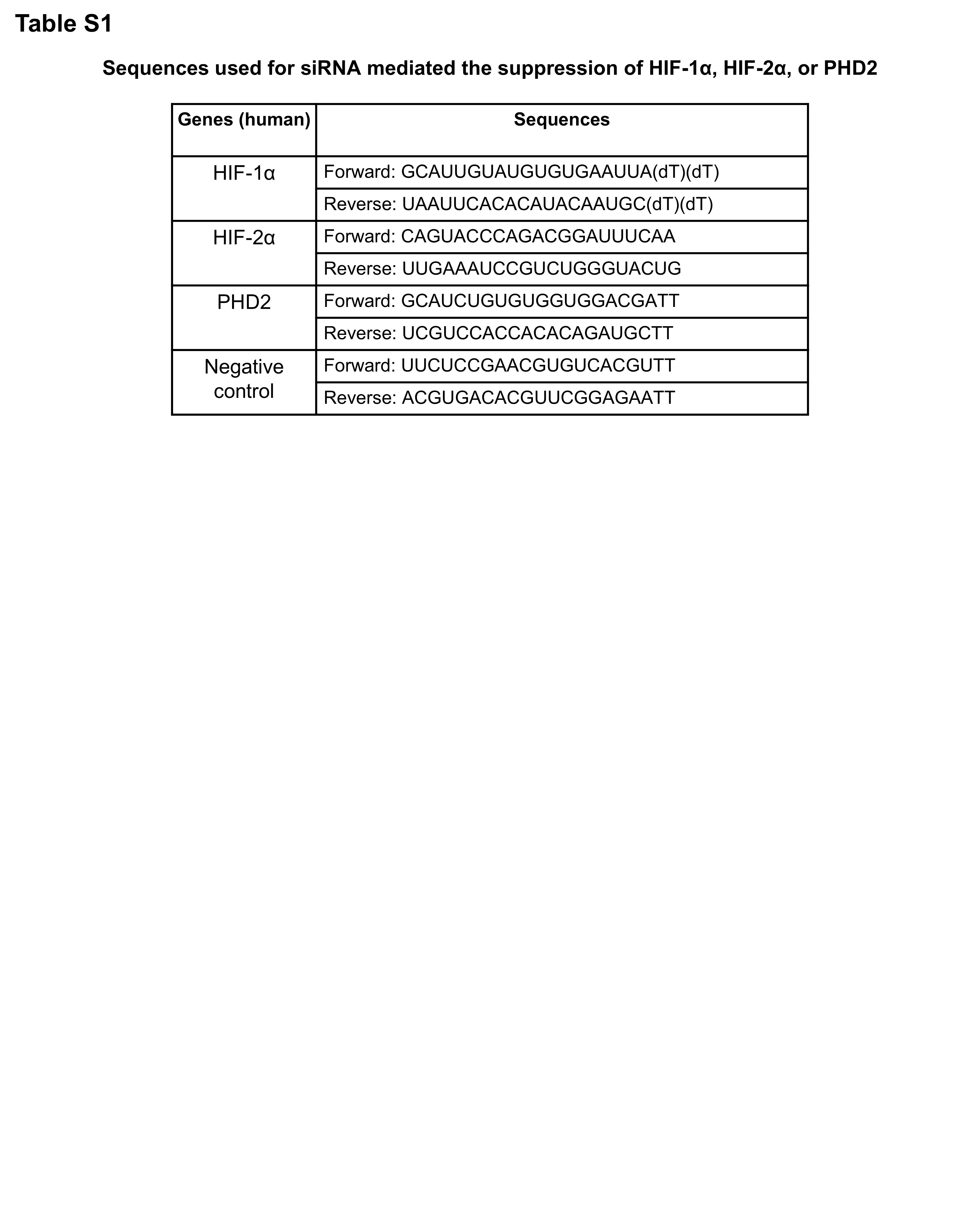
**

The table lists the sense and antisense sequences of siRNAs targeting human HIF-1α, HIF-2α, and PHD2. A non-targeting siRNA (siNC) was used as a negative control.


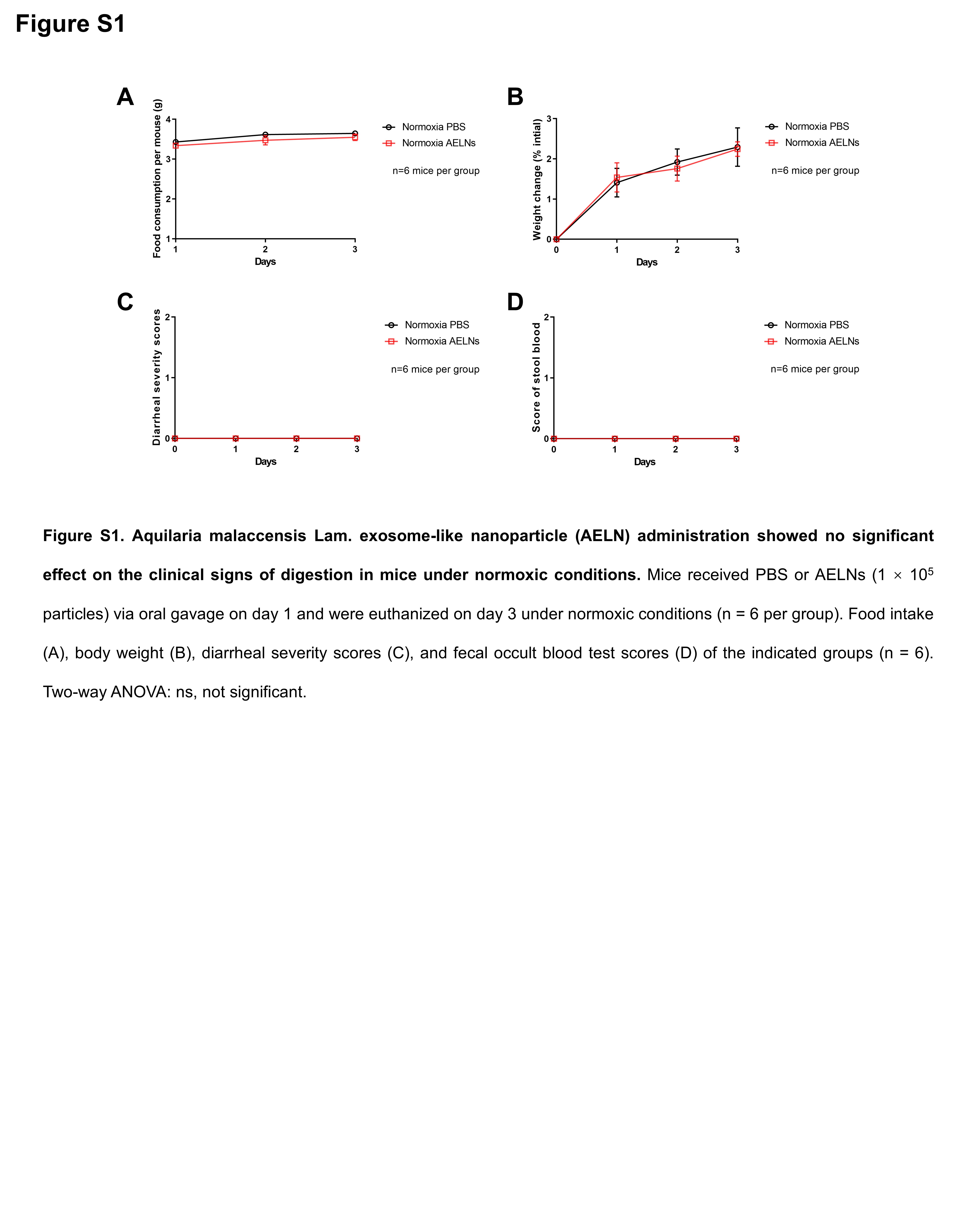


**Supplementary figure S1. Aquilaria malaccensis Lam. exosome-like nanoparticle (AELN) administration showed no significant effect on the clinical signs of digestion in mice under normoxic conditions.** Mice received PBS or AELNs (1 × 10^5^ particles) via oral gavage on day 1 and were euthanized on day 3 under normoxic conditions (n = 6 per group). Food intake (A), body weight (B), diarrheal severity scores (C), and fecal occult blood test scores (D) of the indicated groups (n = 6). Two-way ANOVA: ns, not significant.





**Supplementary figure S2. AELNs partially restored gut microbiota in gastric and small intestinal contents altered by hypoxia.** C57BL/6 mice were housed in a hypoxic chamber for 3 days (n = 6 mice in each group). AELNs (5 × 10^4^ particles) or PBS was administered on day 1, and the mice were euthanized on day 3. The alpha diversity of microbial communities in gastric (A) and small intestinal (B) contents (n = 6). Composition of microbial phyla and genera in gastric (C) and small intestinal (D) contents (n = 6). The relative abundance of significantly altered microbiota in gastric (E) and small intestinal (F) contents (n = 6). Data are presented as the mean ± standard error of the mean (SEM). One-way ANOVA: **P* < 0.05, ***P* < 0.01, ****P* < 0.001, *****P* < 0.0001 as indicated; ns, not significant.


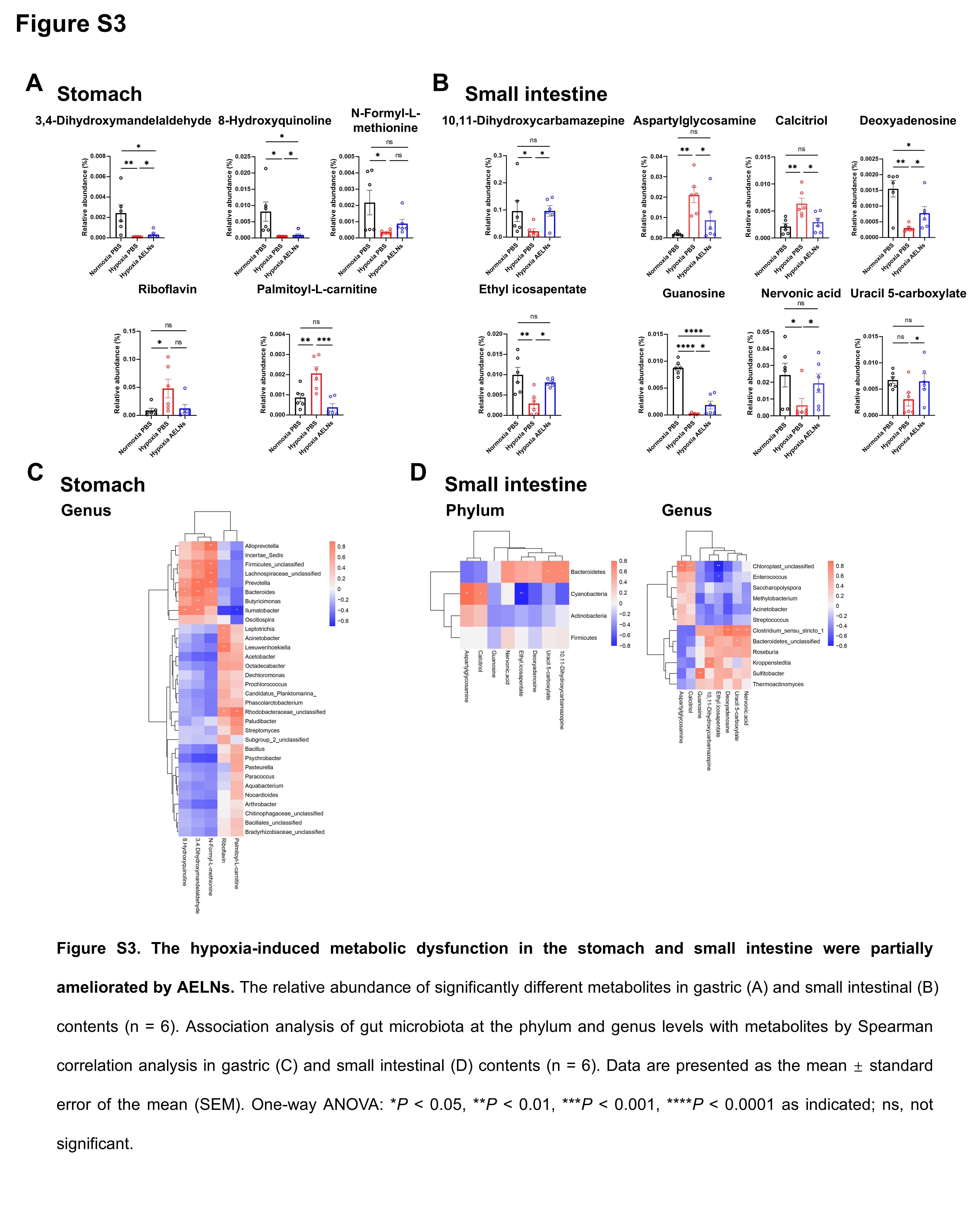


**Supplementary figure S3. The hypoxia-induced metabolic dysfunction in the stomach and small intestine were partially ameliorated by AELNs.** The relative abundance of significantly different metabolites in gastric (A) and small intestinal (B) contents (n = 6). Association analysis of gut microbiota at the phylum and genus levels with metabolites by Spearman correlation analysis in gastric (C) and small intestinal (D) contents (n = 6). Data are presented as the mean ± standard error of the mean (SEM). One-way ANOVA: **P* < 0.05, ***P* < 0.01, ****P* < 0.001, *****P* < 0.0001 as indicated; ns, not significant.


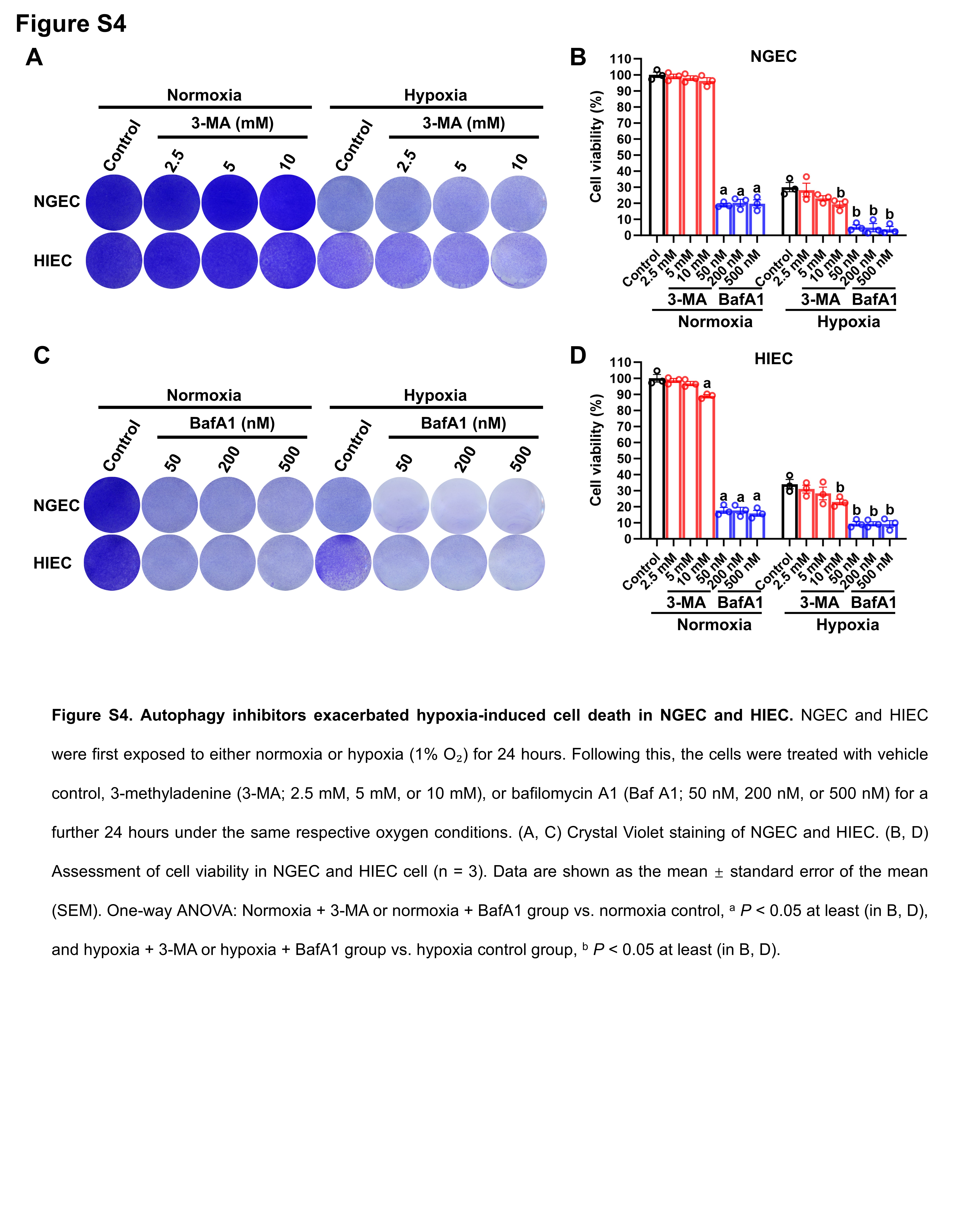


**Supplementary figure S4. Autophagy inhibitors exacerbated hypoxia-induced cell death in NGEC and HIEC.** NGEC and HIEC were first exposed to either normoxia or hypoxia (1% O₂) for 24 hours. Following this, the cells were treated with vehicle control, 3-methyladenine (3-MA; 2.5 mM, 5 mM, or 10 mM), or bafilomycin A1 (BafA1; 50 nM, 200 nM, or 500 nM) for a further 24 hours under the same respective oxygen conditions. (A, C) Crystal Violet staining of NGEC and HIEC. (B, D) Assessment of cell viability in NGEC and HIEC cell (n = 3). Data are shown as the mean ± standard error of the mean (SEM). One-way ANOVA: Normoxia + 3-MA or normoxia + BafA1 group vs. normoxia control, ^a^ *P* < 0.05 at least (in B, D), and hypoxia + 3-MA or hypoxia + BafA1 group vs. hypoxia control group, ^b^ *P* < 0.05 at least (in B, D).


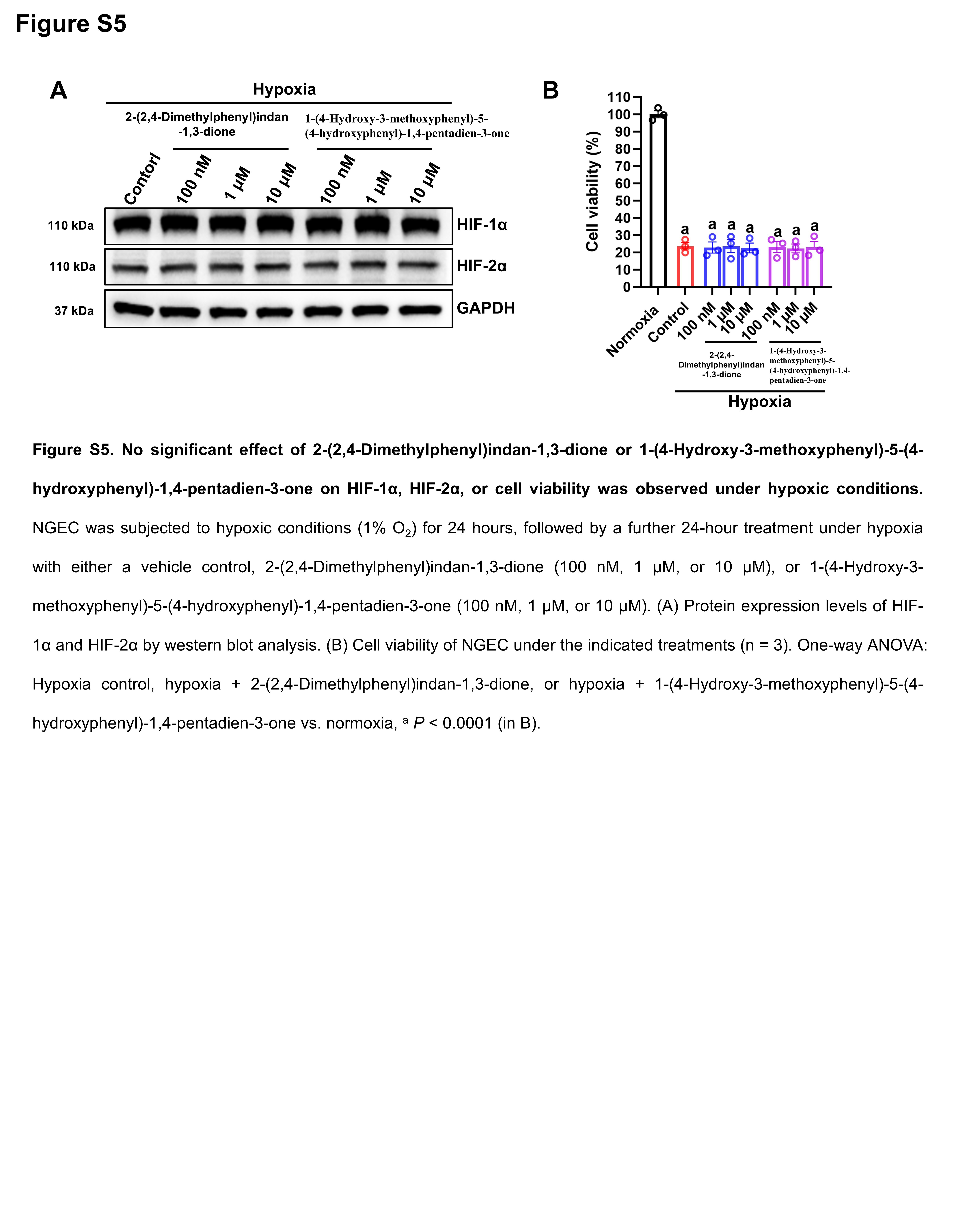


**Supplementary figure S5. No significant effect of 2-(2,4-Dimethylphenyl)indan-1,3-dione or 1-(4-Hydroxy-3-methoxyphenyl)-5-(4-hydroxyphenyl)-1,4-pentadien-3-one on HIF-1α, HIF-2α, or cell viability was observed under hypoxic conditions.** NGEC was subjected to hypoxic conditions (1% O_2_) for 24 hours, followed by a further 24-hour treatment under hypoxia with either a vehicle control, 2-(2,4-Dimethylphenyl)indan-1,3-dione (100 nM, 1 µM, or 10 µM), or 1-(4-Hydroxy-3-methoxyphenyl)-5-(4-hydroxyphenyl)-1,4-pentadien-3-one (100 nM, 1 µM, or 10 µM). (A) Protein expression levels of HIF-1α and HIF-2α by western blot analysis. (B) Cell viability of NGEC under the indicated treatments (n = 3). One-way ANOVA: Hypoxia control, hypoxia + 2-(2,4-Dimethylphenyl)indan-1,3-dione, or hypoxia + 1-(4-Hydroxy-3-methoxyphenyl)-5-(4-hydroxyphenyl)-1,4-pentadien-3-one vs. normoxia, ^a^ *P* < 0.0001 (in B).


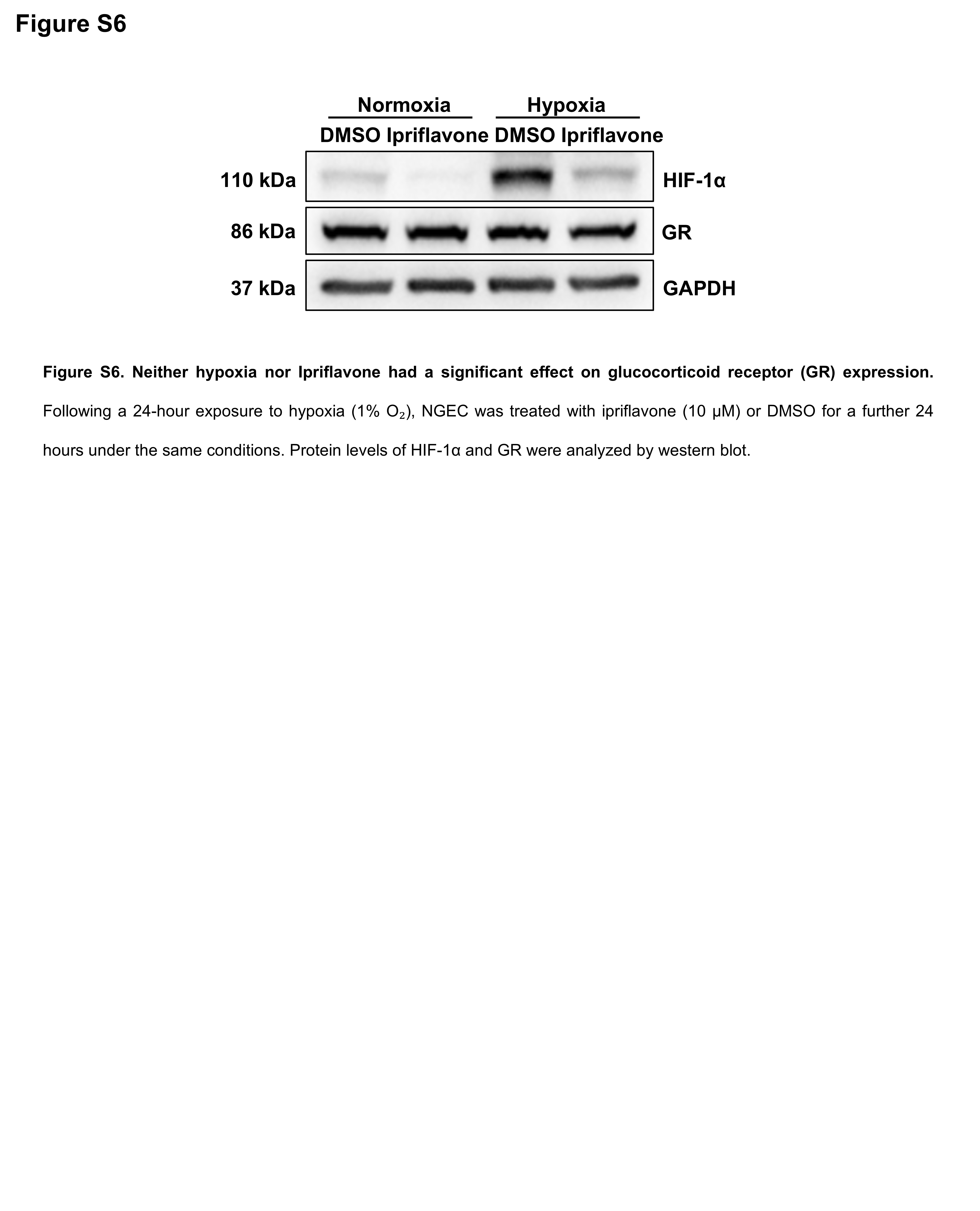


**Supplementary figure S6. Neither hypoxia nor Ipriflavone had a significant effect on glucocorticoid receptor (GR) expression.** Following a 24-hour exposure to hypoxia (1% O₂), NGEC was treated with ipriflavone (10 µM) or DMSO for a further 24 hours under the same conditions. Protein levels of HIF-1α and GR were analyzed by western blot.


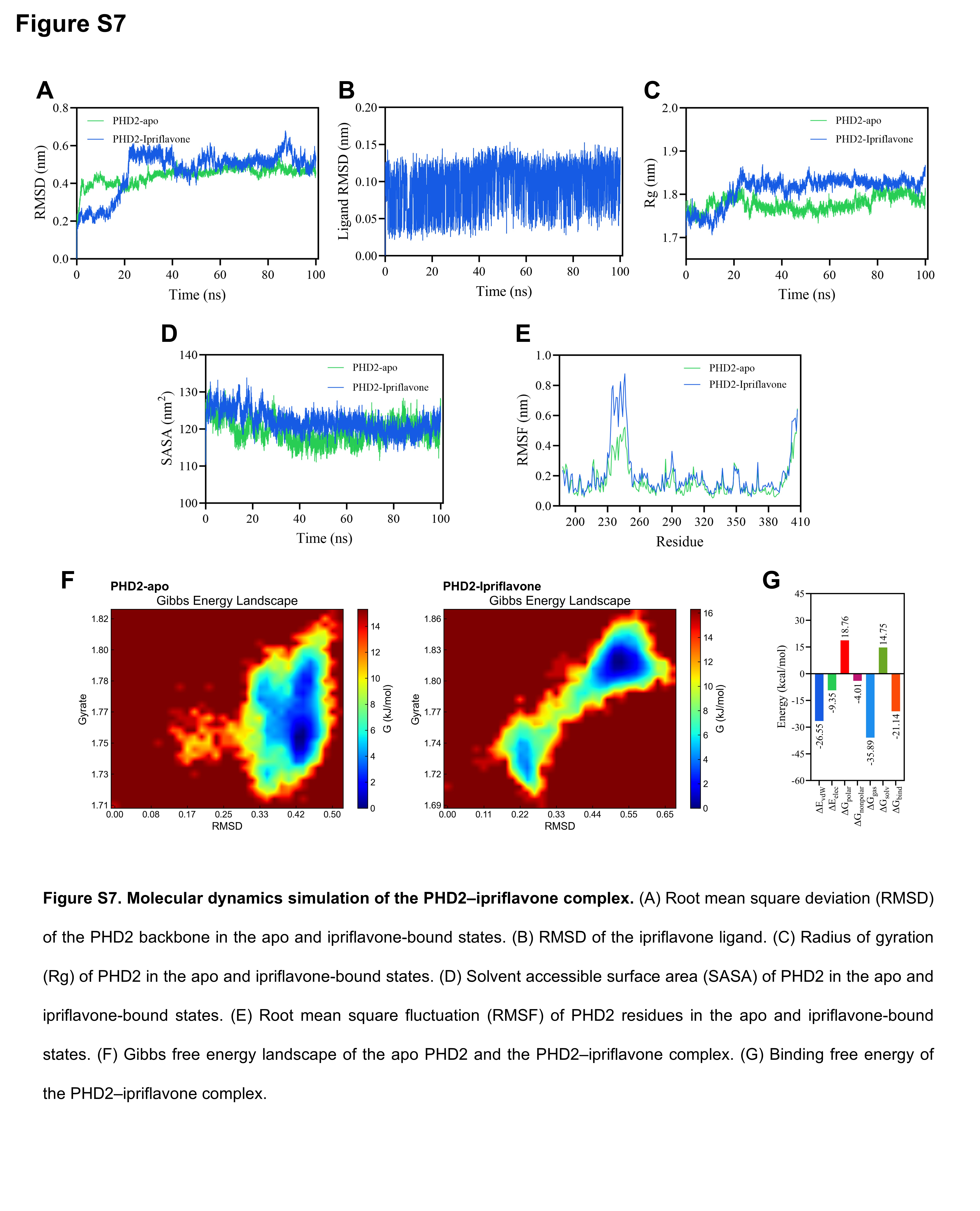


**Supplementary figure S7. Molecular dynamics simulation of the PHD2–ipriflavone complex.** (A) Root mean square deviation (RMSD) of the PHD2 backbone in the apo and ipriflavone-bound states. (B) RMSD of the ipriflavone ligand. (C) Radius of gyration (Rg) of PHD2 in the apo and ipriflavone-bound states. (D) Solvent accessible surface area (SASA) of PHD2 in the apo and ipriflavone-bound states. (E) Root mean square fluctuation (RMSF) of PHD2 residues in the apo and ipriflavone-bound states. (F) Gibbs free energy landscape of the apo PHD2 and the PHD2–ipriflavone complex. (G) Binding free energy of the PHD2–ipriflavone complex.
